# Gα_13_ controls pharyngeal endoderm convergence by regulating E-cadherin expression and RhoA activation

**DOI:** 10.1242/dev.202597

**Published:** 2024-09-30

**Authors:** Bo Hu, Joshua Pinzour, Asmi Patel, Faith Rooney, Amie Zerwic, Yuanyuan Gao, Nhan T. Nguyen, Huaping Xie, Ding Ye, Fang Lin

**Affiliations:** Department of Anatomy and Cell Biology, Carver College of Medicine, University of Iowa, Iowa City, IA 52242, USA

**Keywords:** Pharyngeal endoderm, Convergence and extension, Gα_13_, E-cadherin, RhoA

## Abstract

Pharyngeal endoderm cells undergo convergence and extension (C&E), which is essential for endoderm pouch formation and craniofacial development. Our previous work implicates Gα_13_/RhoA-mediated signaling in regulating this process, but the underlying mechanisms remain unclear. Here, we have used endoderm-specific transgenic and Gα_13_ mutant zebrafish to demonstrate that Gα_13_ plays a crucial role in pharyngeal endoderm C&E by regulating RhoA activation and E-cadherin expression. We showed that during C&E, endodermal cells gradually establish stable cell-cell contacts, acquire apical-basal polarity and undergo actomyosin-driven apical constriction, which are processes that require Gα_13_. Additionally, we found that Gα_13_-deficient embryos exhibit reduced E-cadherin expression, partially contributing to endoderm C&E defects. Notably, interfering with RhoA function disrupts spatial actomyosin activation without affecting E-cadherin expression. Collectively, our findings identify crucial cellular processes for pharyngeal endoderm C&E and reveal that Gα_13_ controls this through two independent pathways – modulating RhoA activation and regulating E-cadherin expression – thus unveiling intricate mechanisms governing pharyngeal endoderm morphogenesis.

## Teaser

**Summary:** Gα_13_ is essential for cell polarity and actomyosin dynamics, regulating pharyngeal endoderm morphogenesis by influencing E-cadherin expression and RhoA activation.

## INTRODUCTION

The endoderm, the innermost germ layer, is crucial for the development of several organs. The gut endoderm, which is located in the posterior region, gives rise to the gastrointestinal tract and associated organs, including the liver and pancreas (Ober et al., 2003; Spence et al., 2011). The pharyngeal endoderm, residing in the head region, contributes to the development of the respiratory tract, pharynx and endoderm-derived glands (Grevellec and Tucker, 2010). Additionally, the pharyngeal endoderm forms endodermal pouches (EPs), which are crucial for guiding the patterning and differentiation of cranial neural crest cells, influencing the development of facial bones and cartilage (Choe and Crump, 2015; David et al., 2002; Graham et al., 2005).

During embryonic development, all germ layers, including the endoderm, undergo convergence and extension (C&E), a process that shapes tissues along the anterior-posterior axis (Zorn and Wells, 2009). In zebrafish, the gut endoderm forms a narrow gut tube (Balaraju et al., 2021), whereas the pharyngeal endoderm initially narrows and then widens to generate a sheet (Ye et al., 2015). After formation of this pharyngeal endoderm sheet, some cells at specific regions migrate laterally to form a series of segmentally arranged EPs (Crump et al., 2004). Thus, the generation of the pharyngeal endoderm sheet is a prerequisite for EP formation. Studies in zebrafish by our group and others reveal that pharyngeal endoderm morphogenesis relies on signaling mediated by the noncanonical Wnt/PCP pathway (Hu et al., 2018; Matsui et al., 2005), and involves a sphingosine-1-phosphate G protein-coupled receptor 2 (S1pr2) and its downstream G protein 13 (Gα_13_) (Ye and Lin, 2013). Notably, pharyngeal endoderm cells do not exhibit planar cell polarity; instead, Wnt/PCP signaling controls the assembly of the extracellular matrix to create the necessary environment for efficient migration of endodermal cells (Hu et al., 2018).

Gα_13_ belongs to the Gα_12_ subfamily of heterotrimeric G proteins and primarily uses the RhoGEF/Rho pathway to control cell shape and migration (Kozasa et al., 2011; Sugimoto et al., 2003), influencing numerous physiological and pathological processes (Worzfeld et al., 2008). Gα_13_ is crucial for blood vessel formation in mice (Offermanns et al., 1997) as well as gastrulation in *Drosophila* (Garcia De Las Bayonas et al., 2019; Kerridge et al., 2016; Kölsch et al., 2007) and zebrafish (Lin et al., 2009). Our previous data reveal that Gα_13_ controls pharyngeal endoderm C&E through RhoGEF/Rho-mediated pathway (Ye and Lin, 2013). Moreover, Gα_13_ is involved in regulating cell-cell adhesion. Both mammalian and zebrafish Gα_13_ physically interact with the intracellular domain of E-cadherin, inhibiting E-cadherin activity and impacting cell-cell adhesion (Lin et al., 2009; Meigs et al., 2002, 2001), suggesting that its role in cell-cell adhesion is not dependent on RhoA. On the other hand, in *Drosophila*, Gα_13_ governs cortical stability in gastrulating cells (Kanesaki et al., 2013), illustrating that its role in cell-cell adhesion is cell-type specific. These findings demonstrate that Gα_13_ acts through different mechanisms to regulate various developmental processes.

Despite existing knowledge, the mechanisms by which Gα_13_ regulates endoderm C&E to generate the endodermal sheet remain unclear. In this study, we addressed this gap using transgenic zebrafish lines in which endoderm cells were specifically labeled. Our findings demonstrate that Gα_13_ is crucial for establishing basal-apical polarity and for mediating actomyosin-driven apical constriction, processes that are crucial for pharyngeal endoderm C&E. These mechanisms facilitate the transition of the endoderm from a monolayer to a two-layer structure, laying the foundation for EP formation. Furthermore, our observations reveal that Gα_13_ deficiency leads to reduced E-cadherin expression, contributing to endoderm defects. Notably, disrupting RhoA function interferes with polarized actomyosin activation but does not affect E-cadherin expression. These data indicate that Gα_13_ regulates pharyngeal endoderm C&E by modulating actomyosin activation and cell-cell adhesion through both RhoA-dependent and -independent pathways, uncovering novel insights into pharyngeal endoderm morphogenesis.

## RESULTS

### The *gna13* zebrafish mutants phenocopy the defects observed in *gna13* knockdown embryos

Our previously published studies demonstrate that injecting morpholino antisense oligos (MOs) to suppress the translation of *gna13a* and *gna13b* genes, which encode Gα_13_a and Gα_13_b in zebrafish embryos, disrupts myocardial cell migration, pharyngeal endoderm convergence and caudal fin development, resulting in cardiac bifida, cardiac edema and tail blistering (Ye and Lin, 2013). To validate these phenotypes, we generated *gna13a* and *gna13b* mutant zebrafish, as described in the Materials and Methods (Fig. S1). Both homozygous *gna13a^−/−^* and *gna13b^−/−^* mutant zebrafish survived to adulthood; thus, we generated *gna13a*^−/−^/*gna13b*^+/−^ and *gna13a*^+/−^/*gna13b*^−/−^ mutants. Offspring obtained from incrossing these mutants exhibited cardiac bifida and cardiac edema (resulting from impaired myocardial cells), as well as tail blistering, like those observed in embryos injected with *gna13a* and *gna13b* MOs (Ye and Lin, 2013).

**Fig. 1.**
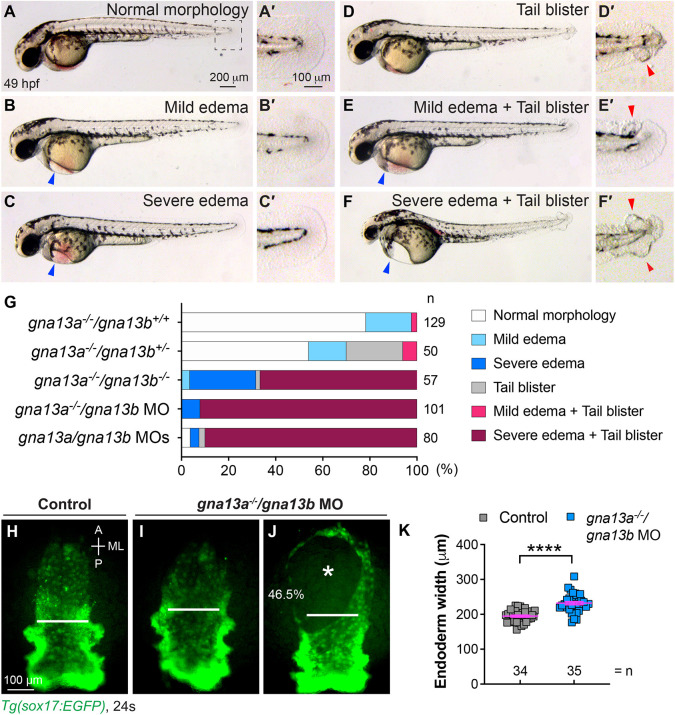
**Phenotypes of *gna13a*^−/−^/*gna13b*^−/−^embryos resemble those observed in *gna13a*/*gna13b* MO-injected embryos.** (A-F′) Bright-field images showing different phenotypic classes of cardiac edema and tail blister in *gna13a/gna13b*-deficient embryos at 49 hpf: normal morphology (A), mild cardiac edema only (B), severe cardiac edema only (C), tail blister only (D), mild cardiac edema and tail blister (E), and severe cardiac edema and tail blister (F). Lateral views. (A′-F′) Zoomed-in images showing the tail region (outlined in A) of embryos in A-F. Blue arrowheads, cardiac edema; red arrowheads, tail blister. (G) The percentage of phenotypic classes from embryos of the indicated genotypes. (H-J) Epifluorescence images of the pharyngeal endoderm in *Tg(sox17:EGFP)* control (H) and *gna13a*^−/−^ embryos injected with *gna13b* MO (I,J) at the 24-somite stage. White lines (equivalent length in embryos) indicate the width of the anterior endodermal sheet; white asterisk indicates the endodermal hole (observed in 46.5% of *gna13a*^−/−^/*gna13b* MO embryos). (K) Average endoderm width in the indicated groups. Data are mean±s.e.m. ****P<0.0001 (unpaired, two-tailed Student's *t*-test).

In this study, we used *gna13a*^−/−^/*gna13b*^+/−^ mutants and evaluated the phenotypes of their offspring based on the severity of cardiac edema and tail blistering at 49 h post-fertilization (hpf), when these features were most pronounced (Fig. 1A-F′). We found that 2.3% of *gna13a*^−/−^/*gna13b*^+/+^ embryos displayed both mild cardiac edema and tail blister, and 19.4% exhibited mild cardiac edema (Fig. 1G). When one copy of *gna13b* was deleted in *gna13a*^−/−^ embryos (*gna13a*^−/−^/*gna13b*^+/−^), these phenotypes became more severe: 6% showed both mild cardiac edema and tail blister, whereas 16% had mild cardiac edema, and 24% exhibited tail blister (Fig. 1G). In *gna13a*^−/−^/*gna13b*^−/−^ embryos, cardiac edema worsened significantly: 67% of embryos showed both severe cardiac edema and tail blister, and 28% of embryos displayed severe cardiac edema (Fig. 1G). Additionally, *gna13a*^−/−^/*gna13b*^−/−^ embryos frequently exhibited smaller eyes (Fig. 1F). These data support our previous findings in *gna13a/gna13b* MOs-injected embryos and demonstrate a redundant functionality between *gna13a* and *gna13b*.

Because *gna13a* and *gna13b* exhibit maternal expression in zebrafish embryos (Lin et al., 2005), we injected *gna13b* MO into *gna13a*^−/−^ embryos (*gna13a*^−/−^/*gna13b* MO) to suppress the translation of both maternal and zygotic *gna13b* RNA. These embryos exhibited more severe phenotypes than those with *gna13a*^−/−^/*gna13b*^−/−^ embryos: all embryos displayed severe cardiac edema and 92% also exhibited tail blistering (Fig. 1G). The severity of these embryos was similar to embryos injected with *gna13a* and *gna13b* MOs, in which 90% showed both severe cardiac edema and tail blistering (Fig. 1G). Western blots using a previously characterized antibody (Lin et al., 2009) showed Gα_13_ expression was significantly reduced or undetectable in *gna13a/gna13b*-deficient embryos (Fig. S1G).

To assess pharyngeal endoderm defects in *gna13* mutants, we crossed them into the *sox17:EGFP* transgenic line, where the endoderm is labeled with EGFP (Ye and Lin, 2013). Both *gna13a*^−/−^/*gna13b*^−/−^ and *gna13a*^−/−^/*gna13b* MO embryos displayed endoderm defects characterized by a wider endoderm sheet that contained holes (Fig. 1H-K and not shown). Next, we investigated whether overexpressing Gα_13_ could rescue the defects in *gna13a*^−/−^/*gna13b*^−/−^ embryos. We injected human *GNA13* RNA into *gna13a*^−/−^/*gna13b*^−/−^ embryos and evaluated pharyngeal endoderm width and the positioning of the myocardium using an MF20 antibody that detects the heavy chain of myosin (Budine et al., 2020). Consistent with our previous data (Ye and Lin, 2013), *gna13a*^−/−^/*gna13b*^−/−^ embryos displayed a wider endoderm and cardiac bifida at 26 hpf; however, these defects were almost completely ameliorated by the expression of human Gα_13_ (Fig. S2A-F). Similarly, expressing human Gα_13_ also reduced the severity of cardiac edema and tail blistering in *gna13a*^−/−^/*gna13b* MO embryos (Fig. S2G). Collectively, our data suggest that *gna13a* and *gna13b* mutants are functionally null and that their phenotypes resemble those of MO-injected embryos. In the context of this study, we used *gna13a^−/−^*/*gna13b^−/−^*, *gna13a^−/−^*/*gna13b* MO or *gna13a/gna13b* MO-injected embryos.

### Gα_13_ is required for morphological changes of endoderm cells during C&E

Distinct changes in the width of the pharyngeal endoderm have previously been observed throughout segmentation, occurring in three phases: rapid narrowing during the 6- to 14-somite stage (ss), slow narrowing at 14-16 ss and moderate widening at 17-22 ss (Ye et al., 2015). These findings suggest dynamic morphological transformations occur in the endoderm during development. In embryos lacking Gα_13_, the pharyngeal endoderm is widened throughout segmentation (Ye et al., 2015).

**Fig. 2.**
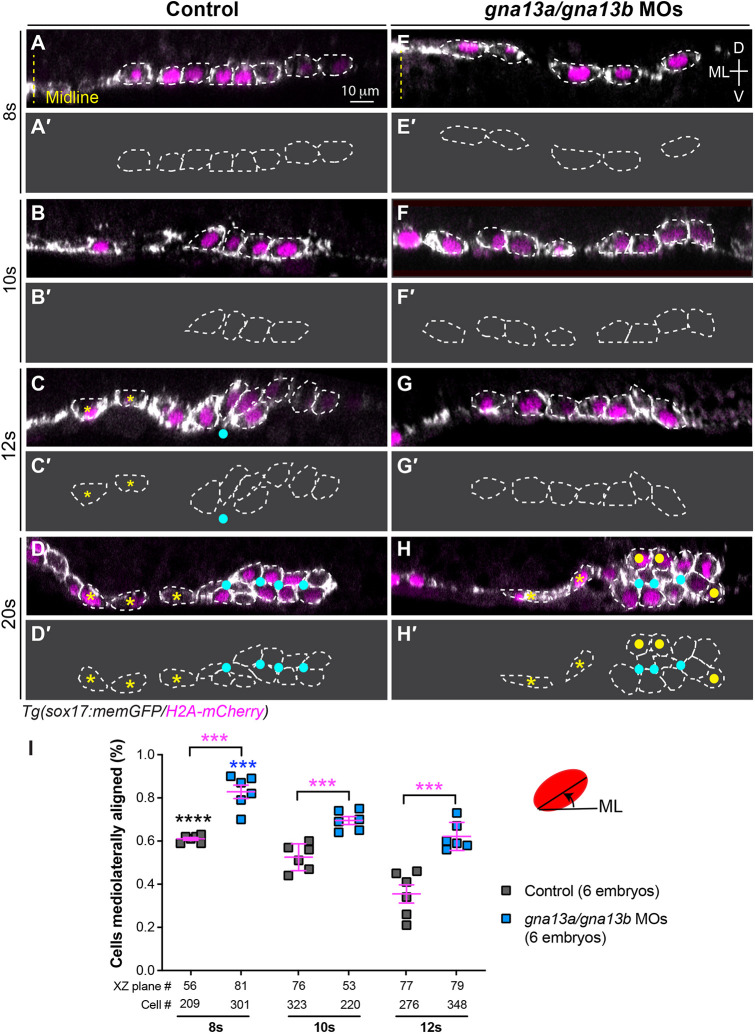
**Gα_13_ regulates pharyngeal endoderm organization during endoderm C&E.** (A-H) *XZ* images of confocal *z*-stacks taken in the anterior region of the pharyngeal endoderm in control (A-D) and *gna13a*/*gna13b* MOs-injected (E-H) embryos at the indicated stages. Some cells are outlined with white dashed lines. (A'-H′) Outlines of some of the endodermal cells in A-H. Yellow dashed line, midline; yellow asterisks, single layer endodermal cells; cyan dots, apical centers; yellow dots, cells that failed to meet at the rosette center. ML, mediolateral; D, dorsal; V, ventral. (I) Percentage of cells in which the longitudinal axis was oriented ±20° relative to the ML embryonic axis in embryos in A-H. Data are mean±s.e.m. ***P<0.001 (magenta; unpaired, two-tailed Student's *t*-test between control and *gna13a/gna13b* MOs-injected embryos). ****P<0.0001 (black; one-way ANOVA analyses for control embryos at different stages); ***P<0.001 (blue; one-way ANOVA analyses for *gna13a/gna13b* MOs-injected embryos at different stages).

To understand the cellular mechanisms of endoderm convergence, we used a transgenic *Tg(sox17:memGFP/H_2_A-mCherry)* zebrafish line in which GFP labels the plasma membrane and mCherry labels nuclei in endoderm cells (Hu et al., 2018). We performed confocal imaging on embryos fixed from 8 ss to 20 ss, capturing dramatic alternations in endodermal width. Analyses of *xy* images from *z*-stacks showed significant changes in endodermal cell shape and orientation in the lateral regions (Fig. 2A-D′).

In control embryos, at 8-10 ss, endodermal cells transitioned from a flat monolayer to cuboidal shapes, initially elongated mediolaterally (Fig. 2A,A′). By 10 ss, they were oriented dorso-ventrally (Fig. 2B,B′). By 12 ss, some cells further elongated to form a rosette structure with a common point (cyan dot, Fig. 2C,C′). Orientation analyses showed that at 8 ss, 61% of cells were aligned within ±20° of the mediolateral (ML) axis, decreasing to 53% at 10 ss and 36% at 12 ss (Fig. 2A-C′,I, Fig. S3A-C), indicating that more cells elongated dorso-ventrally over time. By 20 ss, the rosettes matured and elongated with multiple apical centers (Fig. 2D,D′). These morphogenetic changes convert the endoderm from a monolayer to a two-layer structure. Notably, rosette structures were only observed in the lateral regions of the endoderm, whereas a single layer was maintained in the middle region (yellow asterisks, Fig. 2C-D′,H,H′). Similar changes were observed at earlier stages in the posterior region (not shown), suggesting a posterior-to-anterior progression of these morphogenetic events.

In *gna13a/gna13b*-deficient embryos, 83% of endodermal cells aligned within ±20° of the ML axis at 8 ss (Fig. 2E,E′,I, Fig. S3D). At 10 ss and 12 ss, 70% and 62% of cells, respectively, remained mediolaterally elongated (Fig. 2F-G′,I, Fig. S3E,F), indicating impairment in their cell shape changes. Although rosettes still formed at 20 ss, they appeared disorganized, with cells being less elongated and failing to meet at the rosette center (yellow dots, Fig. 2H,H′). These defects suggest that Gα_13_ is crucial for proper endoderm cell shape changes necessary for rosette formation during convergence.

### Gα_13_ is required for establishing cell-cell contacts and cell shape changes in endoderm cells during C&E

**Fig. 3.**
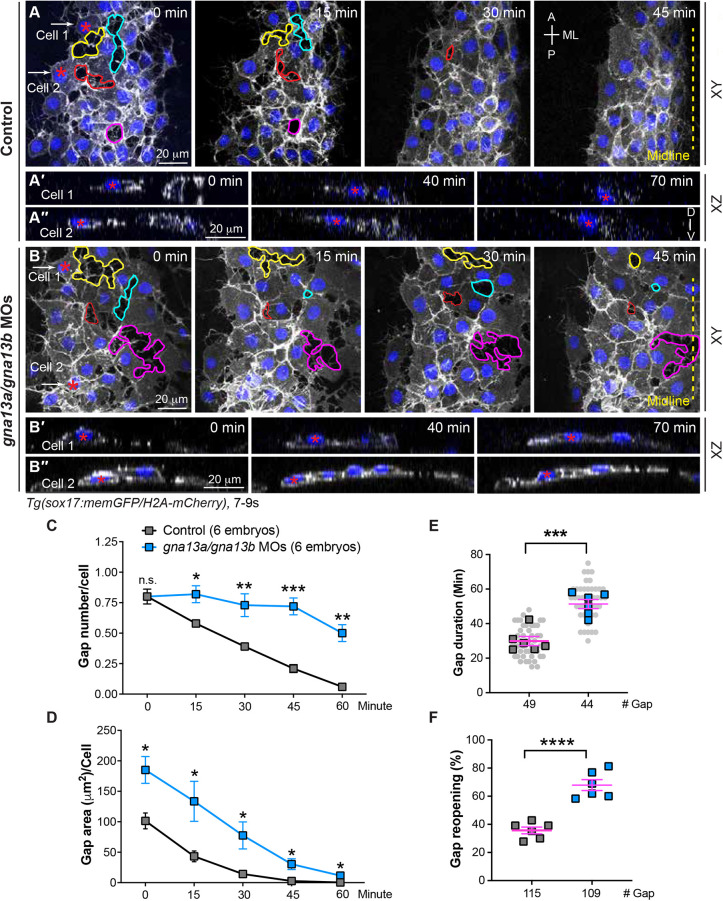
**Gα_13_ regulates stable cell-cell contacts and proper changes of endodermal cell shape during endoderm C&E.** Confocal time-lapse experiments were conducted at the most lateral and anterior regions of pharyngeal endoderm in the indicated embryos during 7-9 ss. (A,B) Snapshots of confocal *z* projections in the *xy* view, taken at different time points from Movie 1, illustrating the morphology of endodermal cells at various time points. Gaps between endodermal cells are outlined, and the same gap over time is outlined by the same color. (A'-B″) Confocal images of *xz* planes of two representative cells (cell 1 and cell 2), captured at regions marked by white arrows in A and B, from Movie 2, illustrating the orientation of the nuclei of two cells at the indicated time-points. (C-F) The average number (C), area (D), duration (E) and reopening frequency (F) of the gaps in endodermal cells in the indicated embryos at different time points. (E) Data from all embryos (squares) and all *xy* images (gray circles) are superimposed. A, anterior; P, posterior; ML, mediolateral; D, dorsal; V, ventral. Yellow dashed line, midline. Data are mean±s.e.m. n.s. (not significant), *P*>0.05; *P<0.05; **P<0.01; ***P<0.001; ****P<0.0001 (unpaired, two-tailed Student's *t*-test).

To investigate how Gα_13_ signaling influences endoderm cell behavior, we conducted time-lapse imaging on the endoderm. Using the head as a landmark, we focused on the most lateral region of the anterior endoderm sheet at 7-9 ss. Our analyses revealed that, in control embryos, endoderm cells migrated towards the dorsal side, which caused the endoderm sheet to become narrower. Notably, at 7 ss, gaps between endoderm cells were visible but gradually shrank and closed as cell-cell junctions formed over time (Fig. 3A). By 9 ss, very few gaps were present, stable cell junctions were established and endodermal cells migrated as a cohesive sheet towards the midline (Fig. 3A, Movie 1). We also tracked the *xy* planes of some cells at the most lateral region of the endoderm over time. Consistent with results in the fixed embryos shown in Fig. 2, we found that endoderm cell nuclei were initially elongated mediolaterally and later tilted toward the ventral side after 70 min (the same cell labelled with magenta asterisks was tracked, Fig. 3A′,A′′, Movie 2).

In *gna13a*/*gna13b*-deficient embryos, endoderm cells also migrated toward the dorsal side, and the endoderm sheet became narrower. However, we observed significantly larger and more frequent gaps between the endodermal cells. Furthermore, these gaps appeared to persist for an extended duration, with a notable number still present by 9 ss (Fig. 3B, Movie 1). Notably, some gaps reappeared after closure (arrows, Fig. 3B, Movie 1). For example, the gap marked by the cyan line gradually diminished at the 15 min time point but reopened at 30 min and had closed again by 45 min. Meanwhile, the gap marked by the yellow line remained open at 45 min (Fig. 3B).

To better understand these defects, we tracked the timelapse movies and quantified the number, area and duration of the gaps. First, we took a snapshot of the movies every 15 min, outlined all gaps and calculated the area of each gap. The average number and area of the gaps were determined by the total cell number in each image. Our analyses revealed that, in control embryos, the average number of gaps decreased over time from 0.8 gaps per cell at 7 ss to 0.06 gaps per cell at 9 ss. In contrast, *gna13a/gna13b*-deficent embryos had 0.84 gaps per cell at 7 ss, but the gap number remained significantly higher throughout the movies, and they had 0.52 gaps per cell by 9 ss (Fig. 3C). Similarly, the average gap area per cell was considerably larger in *gna13a/gna13b*-deficent embryos (Fig. 3D). Second, we randomly tracked a few gaps in each embryo throughout the entire movie and determined the duration of the gaps, excluding those that were not closed by the end of movie. We found that gaps in *gna13a/gna13b*-deficent embryos were open significantly longer than those in control embryos (51 min versus 30 min, Fig. 3E). Finally, to determine the frequency of gaps that reopened, we tracked all gaps throughout the entire movie to identify those that reopened after closing. Our findings showed that 36% of gaps in control embryos reopened, whereas this occurred in 68% of gaps in *gna13a/gna13b*-deficent embryos (Fig. 3F).

These findings indicate that cell-cell contacts were weak and unstable in *gna13a/gna13b*-deficient endoderm, potentially affecting the collective convergent movement and resulting in a wider endoderm sheet. Weak cell-cell adhesion could also contribute to the formation of holes in the endoderm sheet (white asterisk, Fig. 1J). Furthermore, in *gna13a/gna13b*-deficient embryos, there were no discernible changes in the nuclear orientation of endoderm cells within the same time frame in the *xz* plane images (Fig. 3B′-B′′ and Movie 2). These findings suggest that Gα_13_ plays a crucial role in establishing stable cell-cell contacts and initiating endoderm cell shape changes.

### Gα_13_ is required for efficient apical constriction of the endoderm

To unravel the molecular mechanisms through which Gα_13_ shapes endoderm cell behavior during C&E, we focused on rosette formation, which is a pivotal cellular process driving endoderm C&E. The formation of rosettes relies on myosin activation and the actomyosin network (Harding et al., 2014; Hava et al., 2009). We first investigated whether endoderm cells establish basal-apical polarity, a fundamental requirement for rosette formation. To probe this, we assessed the expression of ZO-1, a tight junction protein predominantly found at the apical region of cells (Ferrari et al., 2008; Levic et al., 2021).

**Fig. 4.**
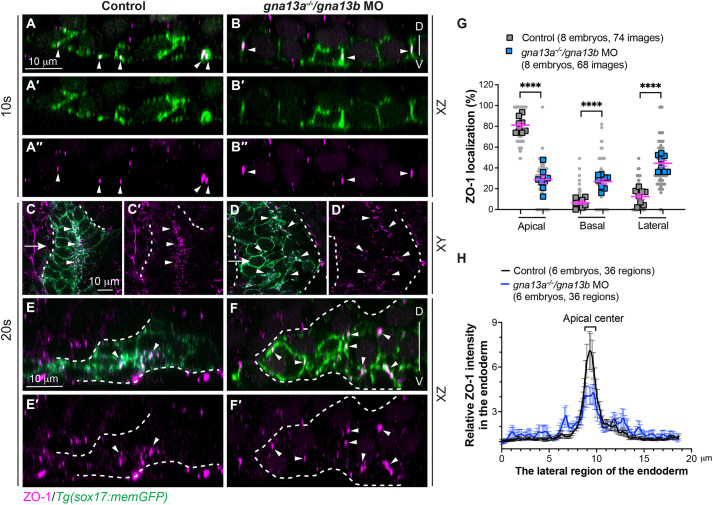
**Gα_13_ is required for efficient apical constriction of endodermal cells.** Whole-mount immunostaining was conducted to determine ZO-1 localization. (A-F′) Confocal images showing ZO-1 localization (magenta, white arrowheads) in pharyngeal endodermal cells (green, labelled by memGFP) in the indicated embryos at 10 ss (A-B″) and 20 ss (C-F′). (A-B″) Confocal images of *xz* planes at 10 ss. (C-D′) Single confocal *z*-plane in *xy* view. (E-F′) Confocal mages of *xz* planes, captured at the positions marked by the white arrows in C,D. White dashed lines outline the endoderm. D, dorsal; V, ventral. (G) The frequencies of ZO-1 localization in the apical, basal and lateral region of endodermal cells in *xz* planes at 10 ss. Data from all embryos (squares) and all *xz* images (gray circles) are superimposed, with the number of *xz* images and embryos indicated. Data are mean±s.e.m. ****P<0.0001 (unpaired, two-tailed Student's *t*-test). (H) Average relative intensity of ZO-1 expression across the lateral region of the endoderm in *xy* planes at 20 ss. The number of regions of images and embryos analyzed is indicated. Data are mean±s.e.m.

Our confocal imaging uncovered that ZO-1 expression in endoderm cells was initially too faint to be detected at early stages (6-8 ss, data not shown) and gradually intensified over time. At 10 ss, analyses of *xz* planes in confocal images revealed that while the endoderm cells retained their mediolateral elongation, ZO-1 expression manifested as small puncta, primarily located on the ventral side of cells (Fig. 4A-A″). This observation implies that the ventral side of the endoderm corresponds to the apical surface. Quantification demonstrated that 81% of ZO-1-labeled puncta were concentrated in the apical region of endoderm cells, with only 6.5% and 12.5% found in the dorsal and lateral regions of the cells, respectively (Fig. 4G). At 20 ss, rosette structures had formed in the lateral region of the endoderm sheet, accompanied by abundant ZO-1-labeled puncta detected at the apical centers of the rosettes (arrowheads in Fig. 4C,C′,E,E′). Plot analyses on single *xy* confocal planes demonstrated that ZO-1 localization was concentrated in a narrow strip at the apical centers (Fig. 4H). These findings illustrate that, during endoderm C&E, endoderm cells gradually establish basal-apical polarity, leading to a progressive reduction of the apical surface to a central point, a phenomenon referred to as apical constriction. This process ultimately culminates in rosette formation.

In *gna13*-deficient embryos, the ZO-1 localization pattern was disrupted. At 10 ss, ZO-1 expression was no longer confined to the apical region but was evident in lateral and dorsal regions of the endoderm cells (arrowheads, Fig. 4B-B″). Quantification revealed that only 28.7% of ZO-1-labeled puncta were located on the apical side of the endoderm cells, whereas 26.9% and 44.4% were observed in the dorsal and lateral regions of the cells, respectively (Fig. 4G). By 20 ss, the overall expression of ZO-1 was notably weakened: ZO-1-labeled puncta were no longer enriched but instead dispersed throughout the rosettes (arrowheads, Fig. 4D,D′,F,F′). Plot analyses demonstrated that the intensity of ZO-1 expression was lower in the apical center and more widely distributed throughout the endoderm sheet (Fig. 4H). Similar results were observed in *gna13a^−/−^/gna13b^−/−^* embryos obtained from incrossing *gna13a^−/−^/gna13b^+/−^* fish (Fig. S4). These results indicate that Gα_13_ plays a crucial role in establishing proper basal-apical polarity and ensuring efficient apical constriction of endoderm cells during C&E.

Next, we explored how the disrupted basal-apical polarity in pharyngeal endoderm that we observed in *gna13a^−/−^/gna13b^−/−^* embryos impacted EP development and subsequent craniofacial skeleton formation. In wild-type embryos, four EPs formed as distinct epithelial structures by 30 hpf (Fig. S5A). Confocal *xz* imaging revealed that these EPs displayed two layers of cells with elongated rosettes, marked by multiple ZO-1-labeled focal points at their apical regions (arrowheads in Fig. S5C,C′). This observation underscores the importance of rosette formation, driven by apical constriction during early stages of development, for the proper establishment of EPs. Notably, in *gna13a^−/−^/gna13b^−/−^* embryos, four EPs also emerged, but the first two EPs were mis-shapen and less elongated compared with controls (Fig. S5D,E versus S5A,B). Confocal *xz* images showed multiple layers of endoderm cells lacking distinct single apical centers in these Eps, with ZO-1 localization scattered rather than concentrated at the rosette centers (Fig. S5F,F′). Quantification demonstrated that, in control embryos, 77% of ZO-1-labeled puncta were concentrated in the apical region of EPs, while in *gna13a^−/−^/gna13b^−/−^* embryos, 41% were in the apical region (Fig. S5G). These results indicate that the defects in apical polarity observed at earlier stages persist into later stages.

Considering the importance of EP formation in craniofacial development (Gao et al., 2022), we examined day 4 embryos and found that all *gna13a^−/−^/gna13b^−/−^* embryos exhibited a small and short lower jaw (Fig. S6A,B). These jaw anomalies were further confirmed through Alcian Blue staining, which reveals cartilage structures. In mutant embryos, the jaw skeleton was noticeably shortened and widened, and Meckel's (m) cartilage, derived from the first pharyngeal arch (PA), appeared shorter and malformed (Fig. S6C,D). Notably, 59% of *gna13a^−/−^/gna13b^−/−^* embryos were missing one side of Meckel's cartilage. Measurements of the length of Meckel's cartilage and the jaw skeleton supported this observation (Fig. S6E). The first PA is located adjacent to the first EP (Choe and Crump, 2014); thus, it is reasonable to infer that the misshaped EP1 is likely a contributing factor to the craniofacial defects observed in *gna13a^−/−^/gna13b^−/−^* embryos.

### Gα_13_ orchestrates spatial actomyosin activity to regulate endoderm convergence

**Fig. 5.**
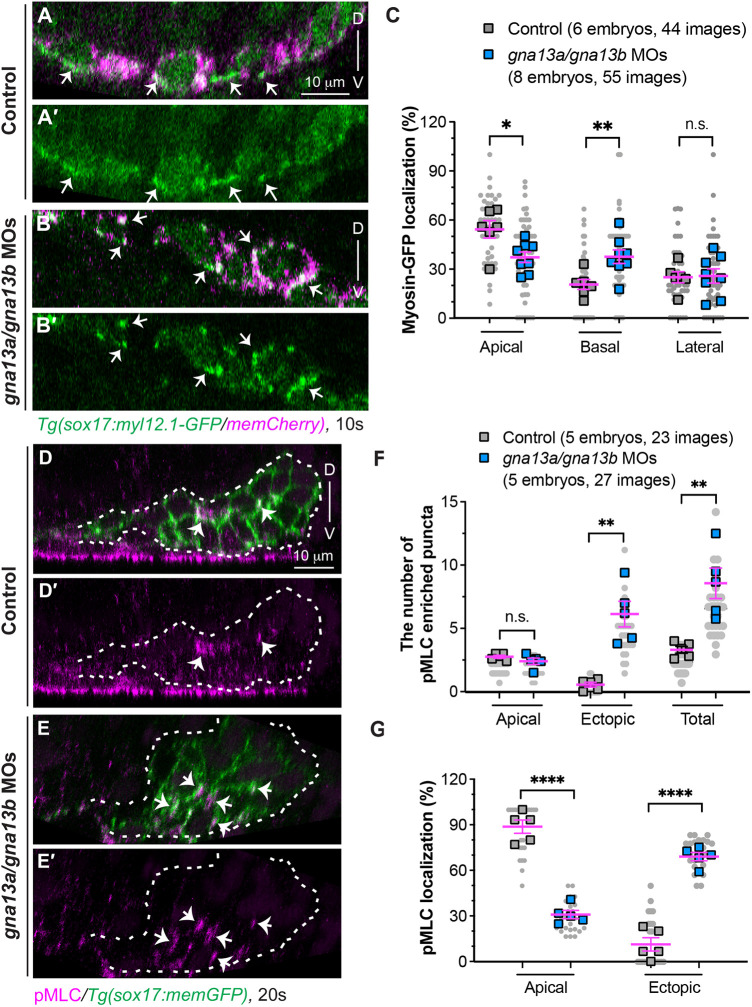
**Gα_13_ controls spatial actomyosin activity to regulate endoderm convergence.** (A-C) Myosin dynamics in the indicated embryos at 10 ss. (A-B′) Confocal images of *xz* planes showing the localization of Myl12.1-GFP in mem-mCherry-labelled endodermal cells (magenta). Arrows indicate enriched Myl12.1-GFP labeled puncta. (C) The frequencies of Myl12.1-GFP labeling in the apical, basal and lateral region of endodermal cells in *xz* planes. (D-G) Expression of pMLC, detected by whole-mount immunostaining, in the indicated embryos at 20 ss. (D-E′) Confocal images of *xz* planes showing the localization of pMLC (magenta) in memGFP-labelled endodermal cells. Arrows indicate pMLC-labeled puncta. White dashed lines outline the endoderm. (F,G) Distribution of pMLC expression. (F) The average number of pMLC-labeled puncta in the apical and ectopic regions, as well as the total number. (G) The frequencies of pMLC-labelled puncta in the apical and ectopic regions in the indicated embryos. Data from all embryos (squares) and all XZ images (gray circles) are superimposed, with the number of *xz* images and embryos indicated. Data are mean±s.e.m. n.s. (not significant), *P*>0.05; *P<0.05; **P<0.01; ****P<0.0001 (unpaired, two-tailed Student's *t*-test). D, dorsal; V, ventral.

We next wanted to determine the molecular mechanisms that drive endoderm apical constriction. We focused on non-muscle myosin II, an evolutionarily conserved actin motor generating contractile forces at the apical site (Martin and Goldstein, 2014; Vasquez et al., 2016). To track myosin II dynamics, we generated a *sox17:myl12.1-GFP* transgenic line, in which myosin light chain 12 is fused to EGFP exclusively in endoderm cells. This transgene serves as a validated marker of actomyosin activation (Behrndt et al., 2012). To evaluate the distribution of myosin-GFP foci, we examined the images of *xz* planes. Similar to ZO-1 localization, in 10 ss control embryos, 54% of myosin-GFP foci were concentrated at the apical region (ventral side) of endoderm cells, with 21% and 25% found in the basal and lateral regions of the cells, respectively (Fig. 5A,A′,C). This indicates contractile actomyosin networks are present in apical regions and generate the necessary cellular forces for apical constriction. In contrast, the apical enrichment of myosin-GFP was lost in *gna13a/gna13b*-deficient embryos. Fewer (37% versus 54%) of myosin-GFP foci were located on the apical side of endoderm cells, and more (37% versus 21%) were found in the basal region, while the labeling in the lateral region was similar to that in control embryos (Fig. 5B-C). This pattern aligns with ZO-1 localization (Fig. 4A-B″,G).

Myosin light chain is activated through phosphorylation by MLC kinase (MLCK) (Ikebe and Hartshorne, 1985), which is a downstream effector of the RhoGEF/RhoA/ROCK (Rho-associated coiled-coil kinase) signaling cascade (Parri and Chiarugi, 2010; Spiegel and Milstien, 2003; Takashima et al., 2008; Vicente-Manzanares et al., 2009). Thus, we reasoned that Gα_13_ signaling could regulate actomyosin contractility by controlling myosin activity. We examined the expression of phosphorylated MLC (pMLC), which was largely localized at the center of rosettes in control embryos, suggesting that myosin-mediated forces drive apical constriction during endoderm convergence (Fig. 5D,D′,F). However, in *gna13a/gna13b* MOs-injected embryos, pMLC expression was not lost but rather diffused in endoderm cells (Fig. 5E,E′). Notably, compared with control embryos, the number of pMLC expressing foci in the apical center was unchanged in *gna13a/gna13b*-deficient embryos, while the number with ectopic expression was significantly increased (Fig. 5F). Thus, the total number of pMLC-expressing foci increased, with a reduced frequency of pMLC localization in the apical center and an increased frequency of ectopic expression (Fig. 5G). These data align with the localization of ZO1 (Fig. 4E,F,H). This indicates that Gα_13_ controls the location in which actomyosin is activated in endoderm cells and, consequently, polarizes contractile forces for endoderm convergence by restricting the activity of signaling molecules.

### E-cadherin expression is diminished in *gna13a/gna13b*-deficient embryos

**Fig. 6.**
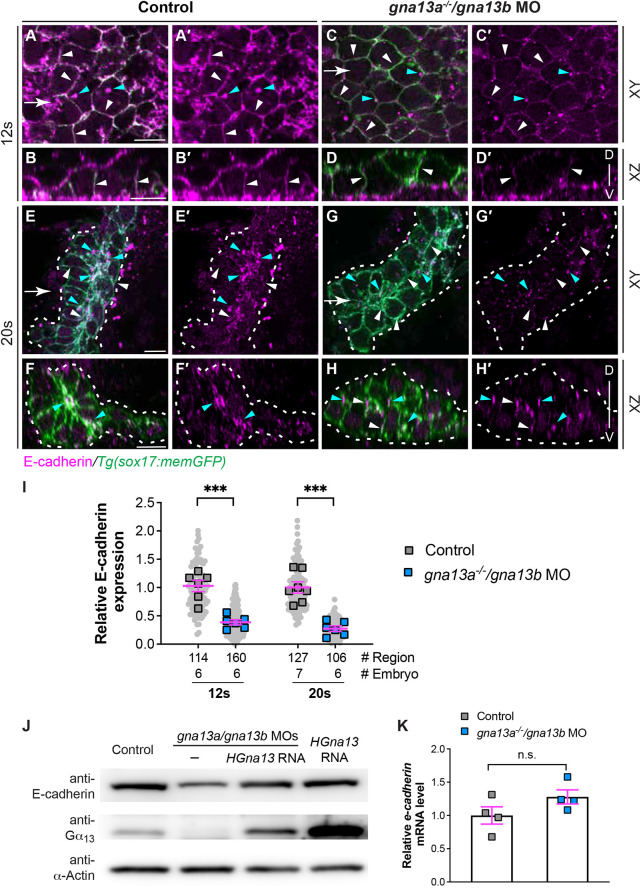
**E-cadherin abundance is reduced in *gna13a/gna13b*-deficient embryos.** (A-H′) Whole-mount immunostaining of E-cadherin localization (magenta) in the indicated embryos. (A,A′,C,C′,E,E′,G,G′) Single confocal *z* plane in the *xy* view. (B,B′,D,D′,F,F′,H,H′) Confocal images of *xz* planes, captured at regions marked by white arrows in A,C,E,G. White arrowheads indicate E-cadherin localization on the plasma membrane of endoderm cells. Cyan arrowheads indicate E-cadherin-enriched puncta in the cytosol of endodermal cells. Dashed lines indicate the endoderm boundary. D, dorsal; V, ventral. (I) Relative intensity of E-cadherin signal on the plasma membrane of endodermal cells at 12 ss in A and C, and in the regions in the endodermal rosettes at 20 ss in E and G in the indicated embryos. Data from all embryos (squares) and regions (gray circles) are superimposed, with the number of regions and embryos indicated. (J) Western blot of E-cadherin and α-actin (loading control) in the indicated embryos at 9 ss. (K) qPCR of *cdh1* mRNA in the indicated embryos at 9 ss. Four experiments were performed. Data are mean±s.e.m. n.s. (not significant), *P*>0.05; ***P<0.001 (unpaired, two-tailed Student's *t*-test). Scale bars: 10 µm.

The presence of excessive gaps observed in time-lapse videos of *gna13a/gna13b*-deficient endoderm cells suggests cell-cell adhesion is impaired (Fig. 3). Thus, we sought to investigate whether *gna13a/gna13b*-deficient embryos exhibited changes in the expression of adhesion molecules. We assessed the expression of E-cadherin and N-cadherin, two major cell adhesion junction proteins that are expressed in zebrafish embryos at an early stage (Dohn et al., 2013). Although we observed no significant alterations in N-cadherin expression in *gna13*-deficient embryos at the transcript level (Fig. S7), a notable reduction in E-cadherin protein expression was detected (Fig. 6). Whole-mount immunostaining of control endoderm cells at 12 ss demonstrated that E-cadherin was primarily localized on the plasma membrane (white arrowheads, Fig. 6A-B′) with some punctate staining in the cytosol of endoderm cells (cyan arrowheads, Fig. 6A-B′). By 20 ss, its localization on the plasma membrane was less prominent (white arrowheads, Fig. 6E-F′), whereas E-cadherin-positive puncta in the cytosol had significantly increased and become enriched in the apical regions of endoderm cells (cyan arrowheads, Fig. 6E-F′). These findings suggest that E-cadherin is actively internalized and becomes enriched in the apical region of endoderm cells during endoderm C&E.

In *gna13a^−/−^*/*gna13b* MO embryos, E-cadherin was also localized on the plasma membrane of endoderm cells at 12 ss, but at significantly lower levels than control embryos (Fig. 6A-D′). The reduced detection of E-cadherin persisted at 20 ss and E-cadherin-bearing puncta in the apical region were much smaller and less pronounced (Fig. 6E-H′). Quantitative analysis revealed a 62% and 73% decrease in the fluorescence intensity of E-cadherin on the endoderm cell membrane of *gna13*-deficient embryos at 12 ss and 20 ss (Fig. 6I). Western blotting using whole-embryo lysates further supported this decrease in E-cadherin expression (Fig. 6J). Overexpressing human Gα_13_ restored E-cadherin expression (Fig. 6J). However, it is noteworthy that the *cdh1* mRNA level detected by qRT-PCR was not altered in *gna13-*deficient embryos (Fig. 6K). The presence of E-cadherin was also reduced in *gna13a^−/−^/gna13b^−/−^* embryos obtained from incrossing of *gna13a^−/−^/gna13b^+/−^* mutant fish (Fig. S8). Collectively, these findings indicate that Gα_13_ regulates E-cadherin expression in a post-transcriptional manner and that disrupting this regulation is linked to endodermal defects.

### Reduced E-cadherin expression contributes to C&E defects in *gna13a/gna13b*-deficient embryos

**Fig. 7.**
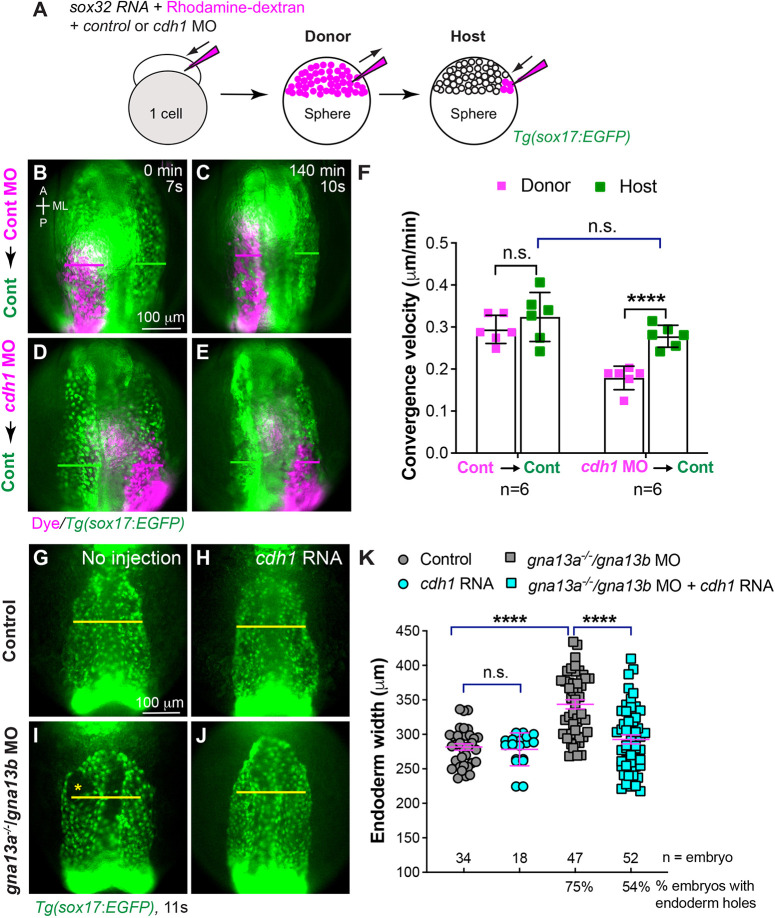
**E-cadherin is responsible for endodermal C&E defects in *gna13a/gna13b-*deficient embryos.** (A) Schematic diagram illustrating endoderm transplantation procedure. Donor embryos were injected with *sox32* RNA (which confers endodermal identity to all cells), rhodamine-dextran (a lineage tracer) and a control *p53* MO or MOs targeting *cdh1* and *p53* at the one-cell stage. At the sphere stage, about 50 donor cells were transplanted into host *Tg(sox17:EGFP)* embryos (endoderm is labeled with EGFP). (B-E) Snapshots from Movie 3 showing the pharyngeal EGFP-labelled endoderm in control *Tg(sox17:EGFP)* hosts transplanted with rhodamine-labeled donor cells (magenta) at the beginning (0 min, 7s) and end (140 min, 10s) of the movie. (B,C) Control MO-injected cells. (D,E) *cdh1* MO-injected cells. Magenta and green lines indicate the widths of the donor and host endodermal sheets, respectively. Dorsoanterior view with anterior upwards. A, anterior; P, posterior; ML, mediolateral. (F) Average convergence velocity of donor and host endoderm in indicated embryos in B-E with the number of embryos indicated. Data were generated from four experiments. (G-J) Epifluorescence images of the pharyngeal endoderm in fixed *Tg(sox17:EGFP*) embryos at 11 ss that were uninjected or injected with *cdh1* RNA. Yellow asterisks indicate endodermal holes. Yellow lines (equivalent length) indicate the width of the pharyngeal endoderm sheet. Dorsoanterior view with anterior upwards. (K) Average width of the pharyngeal endoderm in the indicated embryos shown in G-J, from three experiments. The number of embryos analyzed in each group is indicated. Data are mean±s.e.m. n.s. (not significant), *P*>0.05; ****P<0.0001 (unpaired, two-tailed Student's *t*-test).

To understand how *cdh1*-deficient endoderm cells migrate in a wild-type environment, we conducted endoderm transplantation experiments (Fig. 7A). Donor embryos were injected with a *sox32* RNA to induce endoderm identity, rhodamine-dextran to trace donor cells and a validated *cdh1* MO to suppress E-cadherin translation (Babb and Marrs, 2004). These donor cells were then transplanted into *Tg(sox17:EGFP)* host embryos. Time-lapse experiments were conducted on host embryos, with transplanted donor cells on one side of the anterior endoderm and the opposite side serving as an internal control (Movie 3).

Transplanted cell populations with control MOs migrated to the midline at a pace similar to wild-type host cells, and their final sheet width matched that of the host endoderm populations (Fig. 7B,C,F). In contrast, *cdh1*-deficient cells transplanted in the same environment exhibited significantly slower convergence, resulting in a wider endoderm sheet compared with the wild-type side of the embryo (Fig. 7D-F). These results indicate that E-cadherin is essential for pharyngeal endoderm convergence, resembling the defects observed in *gna13a/gna13b*-deficient embryos.

The reduced expression of E-cadherin in *gna13a/gna13b*-deficient embryos suggested this may contribute to the observed endoderm defects. Therefore, we conducted a rescue experiment by injecting zebrafish *cdh1* RNA into *gna13a/gna13b*-deficient embryos and evaluated the width of the pharyngeal endoderm sheet. Injection of a low dose of *cdh1* RNA had no significant effect on endoderm width in control embryos (Fig. 7G,H,K), but it did reduce the endoderm sheet width and the occurrence of endoderm holes in *gna13a/gna13b*-deficient embryos (Fig. 7I-K). This suggests that overexpressing E-cadherin successfully rescues endoderm defects in *gna13a/gna13b*-deficient embryos and that reduced E-cadherin expression is partially responsible for defective endoderm morphogenesis observed in these embryos.

### E-cadherin plays a vital role in myosin-mediated apical constriction in endoderm cells

To further understand the influence of E-cadherin on myosin-mediated apical constriction, we considered its role as an adhesion molecule on cell membranes, which could serve as anchor points for myosin-mediated contractions (Martin et al., 2010; Rauzi et al., 2010). E-cadherin-bearing puncta were predominantly located at sites of apical constriction in endoderm cells (Fig. 6E,F), coinciding with areas of enriched pMLC (Fig. 5D,D′). This supports the idea that E-cadherin plays a crucial role in maintaining myosin activation at the apical regions of endoderm cells and influences rosette formation.

**Fig. 8.**
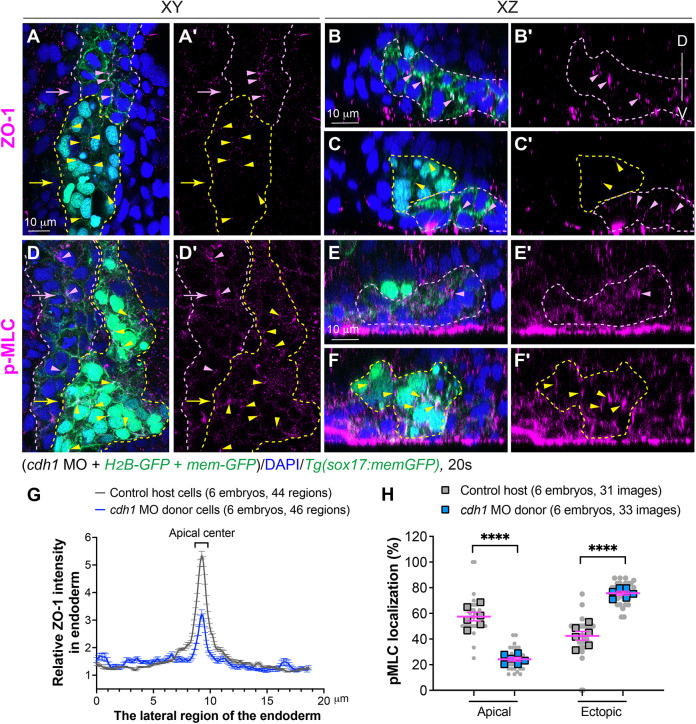
**E-cadherin is crucial for myosin activation and apical polarity of endodermal cells.** Cells from donor embryos co-injected with RNAs encoding *sox32*, *H_2_B-GFP*, *mem-GFP* and *cdh1* MOs were transplanted into control host *Tg(sox17:memGFP)* embryos in which endoderm is labeled with memGFP. (A-F′) Whole-mount immunostaining to detect localization of pMLC and ZO-1 in host embryos at 20 ss. (A,A′,D,D′) Confocal images of a single *z*-plane in *xy* view. (B-C′,E-F′) Images of *xz* planes captured at the positions marked by arrows in A an D. Pink dashed lines indicate host wild-type endoderm cells; yellow dashed lines indicate donor *cdh1*-deficient endoderm cells (nuclei labeled with EGFP). Localization of p-MLC- and ZO-1-expressing puncta in control endoderm cells (pink arrowheads), and in transplanted *cdh1*-deficient endoderm cells (yellow arrowheads). D, dorsal; V, ventral. (G) Average relative intensity of ZO-1 distribution by plot analysis across the lateral region of the endoderm in *xy* planes in donor and host endodermal cell populations. The number of images of *xz* planes and embryos is indicated. Data are mean±s.e.m. (H) The frequencies of pMLC-labelled puncta in the apical and ectopic regions in the indicated embryos. Data from all embryos (squares) and all *xz* images (gray circles) are superimposed, with the number of *xz* images and embryos indicated. Data are mean±s.e.m. ****P<0.0001 (unpaired, two-tailed Student's *t*-test).

To test this hypothesis, we conducted endoderm transplantation with slight modification so that we could target E-cadherin specifically within the endoderm. We injected *H_2_B-GFP* and *mem-GFP* RNAs (as tracers) into donor embryos to label the nuclei and cell membrane of the donor cells, respectively. Subsequently, we evaluated the localization of ZO-1 and pMLC. This assessment was designed to assess the effects of suppressing E-cadherin expression on myosin activation and endoderm organization. Confocal imaging revealed that control endodermal populations in the host formed typical rosette structures, with ZO-1 and pMLC localization predominantly concentrated at the apical centers (Fig. 8A,B,D,E). In contrast, transplanted donor cells injected with a *cdh1* MO failed to form organized endoderm cells with clear rosette formation, and ZO-1 and pMLC labeling showed dispersed patterns within the endoderm (Fig. 8A,C,D,F).

We next conducted quantitative analyses of ZO-1 and pMLC distribution using similar methods as those employed in Figs 4 and 5. Plot analyses on single *xy* confocal planes demonstrated that ZO-1 distribution in *cdh1*-donor endodermal cell populations was less concentrated at the apical centers and more widely distributed compared with the control host endoderm populations (Fig. 8G). Similarly, pMLC localization was scattered in *cdh1* MO-donor endodermal cell populations rather than being enriched at the apical centers, as observed in control host endodermal cells (Fig. 8H). These findings mirror the defects observed in *gna13a/gna13b* deficiency, suggesting that E-cadherin plays a crucial role in regulating spatial actomyosin activation for proper endoderm apical constriction during endoderm C&E.

### Interference with RhoGEF activation disrupts spatial actomyosin activation but does not impact E-cadherin expression

**Fig. 9.**
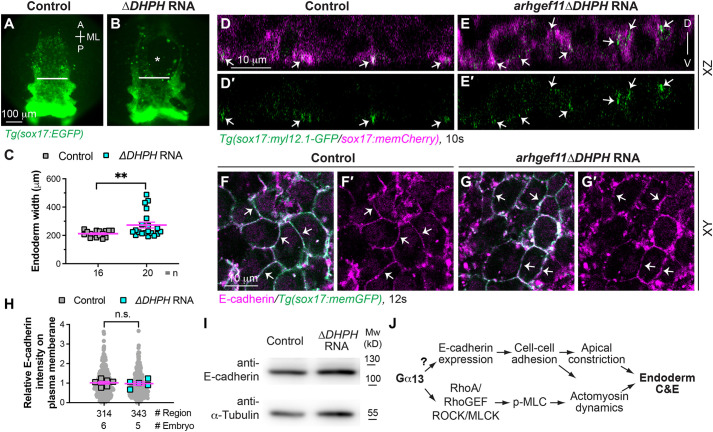
**Interference with RhoGEF disrupts spatial actomyosin activation but does not impact E-cadherin expression.** (A-C) Overexpressing dominant-negative RhoGEF disrupts endoderm C&E. (A,B) Epifluorescence images of pharyngeal endoderm in the indicated embryos. White lines (equivalent length in embryos) indicate the width of the anterior endodermal sheet; white asterisk indicates an endodermal hole. (C) Average endoderm width of the pharyngeal endoderm in the indicated embryos shown in A,B, with the number of embryos indicated. (D-E′) Confocal images of *xz* planes showing localization of Myl12.1-GFP in mem-mCherry-labelled endodermal cells (magenta) in the indicated embryos at 10 ss. White arrows indicate enriched Myl12.1-GFP labelling. (F-I) Overexpressing dominant-negative RhoGEF does not affect E-cadherin expression. (F-G′) Confocal images of a single *xy* plane showing E-cadherin localization (magenta, detected by whole mount immunostaining) in the indicated embryos at 12 ss. White arrows, indicate E-cadherin localization on the plasma membrane of endoderm cells. (H) Relative intensity of E-cadherin expression on the plasma membrane of endoderm cells in the indicated embryos in F,G. Data from all embryos (squares) and regions (gray circles) are superimposed, with the number of embryos and regions indicated. (I) Western blot of the expression of E-cadherin and α-tubulin (loading control) in the indicated embryos at 12 ss. (J) Model illustrating the mechanisms through which Gα_13_ controls endoderm C&E: by regulating both E-cadherin expression and RhoA activation. Data are mean±s.e.m. n.s. (not significant), *P*>0.05; **P<0.01 (unpaired, two-tailed Student's *t*-test).

Our previous study indicates that Gα_13_ regulates myocardial migration and endoderm convergence via a RhoGEF-dependent pathway (Ye and Lin, 2013). Therefore, we investigated whether Gα_13_ influences E-cadherin expression in the endoderm during C&E through RhoGEF. To disrupt RhoGEF activity, we overexpressed a dominant-negative variant of Arhgef11 (Arhgef11ΔDHPH) (Lin et al., 2009). Consistent with our earlier findings (Ye and Lin, 2013), overexpressing Arhgef11ΔDHPH hindered endoderm convergence (Fig. 9A-C). Interestingly, overexpressing Arhgef11ΔDHPH also disrupted the apical localization of myosin-GFP in endoderm cells at 10 ss (Fig. 9D,E), resembling observations made in *gna13a/gna13b*-deficient embryos (Fig. 5A-C). This disruption in RhoGEF activity did not affect E-cadherin expression. E-cadherin expression on the endoderm plasma membrane (Fig. 9F-H) and total protein levels in whole embryo lysates (Fig. 9I) were not different from control embryos. These data suggest that RhoA-mediated signaling exerts an impact on myosin dynamics but does not influence E-cadherin expression. As Gα_13_ regulates both spatial actomyosin activation and E-cadherin expression, our findings suggest that Gα_13_ controls pharyngeal endoderm C&E by regulating actomyosin activation and cell-cell adhesion through both RhoA dependent and independent pathways (Fig. 9J).

## DISCUSSION

### Pharyngeal endodermal C&E involves apical constriction during segmentation

Using specific transgenic lines, our confocal imaging revealed dynamic changes in endodermal cell behavior. During C&E, endodermal cells orient from mediolateral to dorso-ventral directions and form rosettes at lateral regions. These behaviors contract the endoderm along mediolateral axis and elongate it along the anterior-posterior axis. Moreover, analyses of cellular markers showed cells acquire basal-apical polarity during C&E, then constrict apically to form rosettes, transforming the endoderm from a monolayer to a two-layer structure. Once rosettes are fully formed, endodermal convergence halts and endodermal width reaches its narrowest point at 12-14 ss, as reported previously (Ye et al., 2015). Thus, these cellular changes occur during a period when the endoderm undergoes rapid convergence.

The formation of rosettes is fundamental to EP formation (Choe et al., 2013). Our study reveals how these rosettes are formed at earlier stages. Notably, although rosettes form throughout the endoderm sheet, only some regions develop EPs. Moreover, more cells in the EP region participate in rosette formation, elongating structures over time (Choe et al., 2013). Thus, our study uncovers the cellular events that are crucial for endoderm organization and EP formation. However, it remains unclear why rosette structures form specifically in the lateral, rather than the middle, region of the endodermal sheet. Wnt signaling is necessary for EP elongation (Choe et al., 2013), whereas FGF signaling initiates EP formation (Crump et al., 2004; David et al., 2002). Future studies on how these signaling pathways impact endodermal organization, particularly rosette formation, could provide valuable insights into the complex processes initiating EP formation and elongation. Moreover, understanding these mechanisms could illuminate the origins of endoderm-associated diseases.

### Gα_13_-mediated signaling regulates apical constriction for efficient endoderm C&E

Endodermal cells lacking Gα_13_ display impaired basal-apical polarity, leading to randomization of apical markers. This disruption affects the orientation of cells towards the apical side, hinders the formation of rosette structures and ultimately impacts endoderm C&E. This underscores the role of Gα_13_ not only in cell migration but also in establishing basal-apical polarity that is critical for proper cell shape changes during apical constriction.

In *gna13a/gna13b*-deficient embryos, disruption in basal-apical polarity persists in endodermal rosette structures within EPs, affecting proper EP elongation. Given the crucial role of EP development in the migration, patterning, and differentiation of cranial neural crest cells (CNCCs) (Choe and Crump, 2015; Graham et al., 2005; Piotrowski and Nüsslein-Volhard, 2000), these defects likely contribute to cartilage abnormalities observed in *gna13a/gna13b*-deficient embryos at later stages. Similar cartilage defects were also noted in *s1pr2*-deficient embryos (Balczerski et al., 2012), possibly due to comparable endodermal disruptions (Ye and Lin, 2013).

### Gα_13_ controls endoderm C&E by regulating spatial actomyosin activation and E-cadherin expression

Apical constriction primarily relies on activation of actomyosin (Martin and Goldstein, 2014; Vasquez et al., 2016). Our examination of myosin II dynamics revealed concentrated contractile actomyosin networks in the apical regions of endodermal rosettes, which are crucial for apical constriction. Gα_13_ triggers RhoGEF/RhoA/ROCK signaling cascades (Spiegel and Milstien, 2003; Takashima et al., 2008), leading to MLC activation (Parri and Chiarugi, 2010; Vicente-Manzanares et al., 2009), suggesting Gα_13_ influences myosin activity and actomyosin activation. Surprisingly, in the absence of Gα_13_, spatial distribution of actomyosin activation changes whereas overall levels remain unaffected.

Our findings contrast with *Drosophila* studies where the Gα_13_ homolog *concertina* is essential for myosin II expression (Garcia De Las Bayonas et al., 2019; Kerridge et al., 2016). Disparity may stem from the numerous RhoGEFs in vertebrates (Panizzi et al., 2007), allowing myosin activation via alternative pathways (Cook et al., 2014; Newell-Litwa et al., 2015). Additionally, zebrafish Gα_12_, a Gα_12/13_ subfamily member, could facilitate Rho activation (Lin et al., 2005; Suzuki et al., 2003), collectively influencing the actomyosin activation threshold.

The subsequent question is how Gα_13_ regulates spatial activation of actomyosin. The abundance of RhoGEFs in zebrafish poses a challenge in pinpointing which specific RhoGEF is implicated in Gα_13_-mediated endoderm C&E. In *Drosophila*, the distinctive expression patterns of different RhoGEFs may be instrumental in orchestrating the spatial activation of Gα_13_, thereby localizing actomyosin activation and facilitating morphogenetic events (Garcia De Las Bayonas et al., 2019). Despite our attempts to use available antibodies that target Gα_13_, conclusive results were not attained. The development of antibodies against the active status of Gα_13_ or the generation of a zebrafish line in which Gα_13_ is activated by a FRET-based reporter (Mastop et al., 2018) could help address this question.

During segmentation, endodermal cells initially migrate individually before establishing cell-cell contacts and transitioning into collective migration as a cohesive sheet. This progression underscores the pivotal role of cell-cell adhesion in endoderm C&E. Strikingly, in *gna13a/gna13b*-deficient embryos, we observed pronounced abnormalities in cell-cell adhesion within the endodermal cells, which were accompanied by reduced expression of E-cadherin protein. However, we found *cdh1* transcript levels unchanged in the deficient embryos. Thus, the reduced abundance of E-cadherin is due to defects in post-transcriptional events. Supporting this notion, we found that, although E-cadherin protein is uniformly distributed on the plasma membrane of endodermal cells at early stages, it translocates to the cytosol with minimal presence on the plasma membrane and is notably concentrated in the apical center of rosettes. These findings suggest an active endocytosis process for E-cadherin that likely contributes to the regulation of its distribution and ability to facilitate morphogenic changes in endodermal cells during C&E.

It is worth noting that, although *cdh1* transcript levels are not altered in *gna13a/gna13b*-deficient embryos, injecting *cdh1* RNA rescued the defects observed in these deficient embryos. This is likely due to ectopic expression of E-cadherin protein from the injected RNA. Another potential factor could be that synthetic RNAs lack the 5′UTR and 3′UTR elements of endogenous *cdh1* transcript, allowing the injected RNAs to bypass post-transcriptional regulation and increase protein levels of E-cadherin.

Considering that Gα_13_ can bind and interact with E-cadherin, it is plausible that the translocation of E-cadherin to the apical center brings Gα_13_ to this region. This, in turn, could activate downstream molecules, resulting in spatial myosin activation at the rosette center. Indeed, specific targeting of E-cadherin in the endoderm disrupted apical localization of ZO-1 and pMLC, impairing endoderm C&E. Our rescue experiment further demonstrates that diminished expression of E-cadherin is partially responsible for the endodermal defects observed in *gna13a/gna13b*-deficient embryos.

The central question that remains is how Gα_13_ regulates E-cadherin protein expression. E-cadherin dynamics are commonly regulated by diffusion and trafficking, which are controlled by mechanisms including those mediated by small G proteins (de Beco et al., 2012). In *Drosophila*, inhibiting the function of ROCK, a downstream effector of RhoA, suppresses E-cadherin expression (Coravos and Martin, 2016). Additionally, interference with ROCK activity can impede the accumulation of Rab11-expressing vesicles at the apical region, a process that is essential for stabilizing E-cadherin expression (Chen and He, 2022).

Contrary to these findings, our data indicate that overexpressing DN-RhoGEF has minimal impact on E-cadherin expression, suggesting Gα_13_ regulates E-cadherin expression through a RhoA-independent pathway. This aligns with studies in the epithelium of the fly pupal notum, where ROCK is not implicated in E-cadherin expression (Curran et al., 2017). Thus, regulating E-cadherin expression by RhoA/ROCK may be tissue dependent. Subsequent investigations will focus on elucidating the specific mechanism by which Gα_13_ governs E-cadherin expression in the endoderm.

In conclusion, our study elucidates the crucial role of Gα_13_ in orchestrating pharyngeal endoderm C&E, which is crucial for EP formation and craniofacial development. Specifically, we uncover that Gα_13_ independently modulates RhoA activation and E-cadherin expression, providing valuable insight into the intricate mechanisms governing pharyngeal endoderm morphogenesis.

## MATERIALS AND METHODS

### Zebrafish strains and maintenance

Zebrafish were maintained according to animal protocols approved by the University of Iowa Animal Care and Use Committee. Embryos were obtained by natural mating and staged according to morphological criteria or hours post fertilization (hpf) at 28°C or 32°C, unless otherwise specified. The following zebrafish lines were used in this study: AB*/Tuebingen, *Tg(sox17:memGFP/H_2_A-mCherry*) (Hu et al., 2018) and *Tg(sox17:EGFP)* (Mizoguchi et al., 2008).

### Generation of *Tg(sox17:myl12.1-GFP)* zebrafish line and *gna13* mutants

The *sox17:myl12.1-GFP* transgenic construct harbors myosin light chain 12 fused to EGFP and was generated using a Tol2-based Multi-Site Gateway system (Invitrogen) as described previously (Hu et al., 2018; Kwan et al., 2007; Villefranc et al., 2007). The construction involved using the following components: a 5′-entry clone (*p5E-sox17*) containing a *sox17* promoter (Woo et al., 2012), a middle entry clone (*pME*-*myl12.1-GFP*) (Maître et al., 2015), a 3′-entry clone (*p3E*-polyA) and the destination vector *pDestTol2pA2.* At the one-cell stage, AB* embryos were injected with transgene plasmid DNAs (40 pg) and *tol2* mRNA (25 pg), and subsequently screened for GFP expression in the endoderm. GFP-positive embryos identified in this screening were raised as F0 founders and then bred with AB* fish to establish stable lines.

The genetic *gna13a* mutant was created using transcription activator-like effector nucleases (TALENs). TALENs designed to target the first exon of *gna13a* were developed by Dr Keith Joung's laboratory as part of the third solicitation for NIH-funded Resource to Target Zebrafish Genes with Engineered Nucleases (Sander et al., 2011). The Right-TALEN (Addgene plasmid 41263, TAL3089) binds to the sequence TGGAGAGAAGACAATTGG, while the Left-TALEN (Addgene plasmid 41262, TAL3088) binds to TTCCTGCCCTCCCGGACT. Following the confirmation of TALEN plasmid DNAs, TALEN RNAs were synthesized using the mMessage mMachine T7 ULTRA kit (ThermoFisher Scientific, AM1345) and then injected into wild-type embryos. F0 embryos resulting from injection were screened for potential mutations through PCR using primers flanking the potential mutation site (5′-CCGAACATTAAAACCACACCCGT-3′ and 5′-GATCTTCACTAGTTTCTTCACATAGGTCTTCTC-3′). PCR products were either digested with the T7 Endonuclease I (New England Biolabs M0302S, Ipswich, MA) and separated in agarose gels or were analyzed by non-denaturing (native) polyacrylamide gel electrophoresis (PAGE) to detect polymorphisms (Gao et al., 2022). F0 embryos exhibiting potential mutations were raised and stable mutant lines were established. Two mutation alleles, characterized by a −4,+2 indel and a −5 deletion, were confirmed by Sanger sequencing and recovered. The former allele featuring a premature stop codon at V12 (Fig. S1A-C), was used in the present study. The genetic *gna13b* mutant (sa31345) was obtained from the Zebrafish International Resource Center (ZIRC). This allele is characterized by a T to A substitution in the second exon of *gna13b*, resulting in a premature stop codon at L137 (Fig. S1D-F).

To genotype mutants, PCR amplicons were amplified from genomic DNAs and were digested with restriction enzymes for specific patterns. For *gna13a* mutants, an amplicon generated using the following primers (5′-ATCCGCCCATTTGTAACGAA-3′ and 5′-CTGGAGAGAAGACAATTGGGAGTA-3′) was digested with ScaI, producing a 150 bp band from wild-type embryos, and 125 bp and 25 bp bands from mutant embryos. For *gna13b* mutants, an amplicon generated from the following primers (5′-CATGGTGTCGAAAGGGAT-3′, 5′-AATCTCATGTCCCGCTAAAT-3′) was digested with Aju1, which produced bands at 169 bp and 33 bp from wild-type embryos ,and a band at 203 bp from the mutants.

### RNA isolation and quantitative real-time PCR

RNA was extracted from pooled embryos (20-30 embryos at the specified stages) using TRIzol reagent (ThermoFisher Scientific,15596026). cDNAs were synthesized using the iScript Reverse Transcription kit (Bio-Rad Laboratories, 1708840). Real-time PCR was conducted on the Bio-Rad CFX96 Touch Real-Time PCR detection system using the iQ SYBR Green Supermix (Bio-Rad Laboratories, 1708880). The following primers were employed for amplification: *cdh1* (5′-TACACCGAAATCACCTTCAC-3′, 5′-CAGGGACACGCTCAACTC-3′), *cdh2* (5′-GCCATGTCAGCCTGGTTTC-3′, 5′-TCCCATCGGCGTCTATCC-3′) and *eef1a1a* (5′-GAGAAGTTCGAGAAGGAAGC-3′, 5′-CGTAGTATTTGCTGGTCTCG-3′). The relative expression levels of *cdh1* and *cdh2* were determined by normalizing to the expression of *eef1a1a*.

### Cloning *cdh1* and injecting RNAs and morpholinos (MOs)

Full-length zebrafish *cdh1* (NM_131820.1) was cloned from a cDNA library generated from 12 hpf embryos using an overlapping extension PCR strategy. Two sets of primers, each containing overlapping sequences (underlined), were used to amplify the N-terminal (5′-ATATGAATTCAATGGCTTGTGTAACAACTGTGGGA-3′, 5′-TCACAGTGA CTGTGGCAGTTGAAGTAGGCAA-3′) and C-terminal (5′-TTGCCTACTTCAACTGCCA CAGTCACTGTGA-3′, 5′-TATACTCGAGTTAATCCTCTCCTCCTCCATACATGTCC-3′) coding sequences of *cdh1*. In the second round of PCR, the N-terminal and C-terminal amplicons served as templates, and the primers containing the EcoRI and XhoI sites (underlined) (5′-ATATGAATTCAATGGCTTGTGTAACAACTGTGGGA-3′, 5′-TATACTCGAGTTAATCCTCTCCTCCTCCATACATGTCC-3′) were used. Subsequently, the amplicons were cloned into the *pCS2-Myc* vector after digestion with EcoRI and XhoI. All PCRs were carried out using Q5 high-fidelity DNA polymerase (New England Biolabs, M0491S). The constructs were verified by Sanger sequencing.

Capped messenger RNAs were synthesized using the SP6 mMessage mMachine kit (ThermoFisher Scientific, AM1340) and subsequently injected into one-cell embryos. The RNAs encoding the following genes were used at the specified doses: *HGNA13* (90 pg) (Lin et al., 2005), *memGFP* (240 pg), *H_2_B-GFP* (70 pg), *sox32* (250 pg), *arhgef11ΔDHPH* (450 pg) (Lin et al., 2009) and *Myc-Cdh1* (90 pg). Validated morpholino antisense oligonucleotides (MOs) targeting the following genes were employed: *gna13a* (4 ng, 5′-AAATCCGCCATCTTTGTAGTAGCGA-3′) (Lin et al., 2005); *gna13b* (4 ng, 5′-AGGAAATACGCCATCTTTGTGCAAC-3′) (Lin et al., 2005); *cdh1* (6 ng, 5′-AAGCATTTCTCACCTCTCTGTCCAG-3′) (Kane et al., 2005); and *p53* (1.5 ng, 5′-GCGCCATTGCTTTGCAAGAATTG-3′) (Robu et al., 2007). To mitigate potential p53-dependent cell death induced by off-targeting effects of MOs, all MOs were co-injected with the *p53* MO (Robu et al., 2007).

### Endoderm transplantation

Endoderm transplantation was performed using a pneumatic microinjector (Narishige, 16375), as described previously (Hu et al., 2018). Briefly, donor embryos at the one-cell stage were injected with *cdh1* MO and RNA encoding *sox32* (250 pg, to confer an endodermal identity to all cells), along with 0.2% rhodamine-dextran (70,000 MW, lysine-fixable, Invitrogen) or RNAs encoding *mem-mGFP* (240 pg) and *H_2_B-GFP* (70 pg), as lineage tracers. At the sphere stage, 30-50 donor cells were transplanted into the host embryos along the blastoderm margin. Host embryos were screened for rhodamine-labeled donor cells in the pharyngeal endoderm before time-lapse imaging or screened for H_2_B-GFP-labeled cells before immunofluorescence staining.

### Immunofluorescence staining, western blotting and Alcian Blue staining

For immunofluorescence staining, embryos were manually dechorionated and fixed in 1% paraformaldehyde (PFA) at room temperature for 2 h or at 4°C overnight. Subsequently, the yolk of the embryos was manually removed, and embryos were re-fixed in 4% PFA for an additional 2 h at room temperature (Frommelt et al., 2023). For MF20 staining, embryos were fixed in 4% PFA for 2 h at room temperature. In the case of pMLC staining, a phosphatase inhibitor cocktail (MilliporeSigma, 524627) was added to 1% PFA and blocking solution (described below). Permeabilization was carried out in PBS containing 0.5% Triton X-100 for 1 h at room temperature (for embryos before 24 hpf) or in acetone at −20°C for 7 min (for embryos at 32 hpf), followed by two 30 min washes in water.

Immunofluorescence staining was performed in blocking solution (1% BSA, 2% DMSO, 0.1% Triton X-100 in PBS) containing the following primary antibodies: anti-pMLC (1:40, Cell Signaling Technology, 3671S), anti-ZO-1 (1:200, ThermoFisher Scientific, 33-9100), anti-E-cadherin (1:400) (Babb and Marrs, 2004), anti-MHC (1:30, DSHB MF20), and anti-N-cadherin (1:500, Abcam, ab21126). Secondary antibodies used were: Alexa Fluor 647-conjugated donkey anti-rabbit IgG (H+L) (1:300, ThermoFisher Scientific, A-31573), Alexa Fluor 568-conjugated goat anti-mouse IgG (H+L) (1:300, ThermoFisher Scientific, A-11004). Embryos were counterstained with 4′,6-diamidino-2-phenylindole (DAPI, 0.2 µg/ml, ThermoFisher Scientific, D1306) for 15 min and mounted in 90% glycerol/PBS medium containing 0.2% propyl gallate before imaging.

For western blotting, embryos were manually de-chorinated and the yolk was removed following the procedure described previously (Hu et al., 2021). Cell pellets from embryonic tissues were lysed in RIPA buffer, supplemented with equal volume of 2×SDS loading buffer (3 µl/embryo for embryos aged before 24 hpf, 6 µl/embryo for embryos at 52 hpf). Lysates, representing the equivalent of 6-10 embryos, were loaded into polyacrylamide gel for electrophoresis. Immunoblotting was conducted using the following antibodies: anti-Gα_13_ (1:1000) (Lin et al., 2005), anti-E-cadherin (1:4000) (Babb and Marrs, 2004), anti-α-actin (1:1000, DSHB JLA20), anti-α-tubulin (1:1000, Santa Cruz Biotechnology, SC-23948) and anti-N-cadherin (1:500, Abcam, ab21126).

For Alcian Blue staining, embryos exhibiting pronounced edema and tail blistering were selected as representatives of *gna13a*^−/−^/*gna13b*^−/−^ mutants. The embryos were fixed at 5 days post-fertilization (dpf) in 4% PFA at 4°C overnight. Alcian Blue staining was performed using 0.1% Alcian Blue (Sigma-Aldrich) in acid alcohol buffer (0.37% HCl and 70% ethanol), as described previously (Gao et al., 2022).

### Microscopy and time-lapse imaging

For still imaging, both live and fixed embryos were mounted in 2.5% methylcellulose or in 95% glycerol/PBS (for Alcian Blue staining imaging). Epifluorescence images were acquired using a Leica DMI6000 microscope equipped with a 5×/NA 0.15 or 10×/NA 0.3 objective. Bright-field images and images of Alcian Blue staining were captured using a Leica M165FC stereomicroscope with a Leica DFC290 color digital camera. All images were acquired using the Leica LAS X software.

For confocal imaging, embryos were flat-mounted on bridged slides as described previously (Frommelt et al., 2023), and images were captured on laser-scanning confocal inverted microscopes (Zeiss LSM880 or Zeiss LSM980) with EC Plan-Neo 40×/NA 1.3 oil objectives. *Z*-stacks were acquired at optimal intervals using the following settings: zoom 1.5, 1024×1024 pixel, 7 speed and 2 averaging.

For epifluorescence time-lapse imaging, embryos were embedded in 1% low melting-point agarose and mounted into a glass-bottom dish with a dorsal-mount imaging mold, as described previously (Ye and Lin, 2013). Images were taken at 5 min intervals using a 5×/NA 0.15 objective on a Leica DMI 6000 microscope. For confocal time-lapse imaging, embryos were embedded in 1% low melting-point agarose and mounted into a glass-bottom dish (Cellvis, D29-20-1.5-N). Images were captured using a confocal inverted microscope (Zeiss LSM880 or Zeiss LSM980) with a LD C-Apo 40×/NA 1.1 water objective. *Z*-stacks (15-16.5 µm) were acquired to cover the entire pharyngeal endoderm at 1.5 µm intervals every 3-5 min, using the following settings: zoom 1.2, 1024×1024 pixel, 7 speed, and 2 averaging.

### Image analysis

Images of the same type were acquired and processed in Fiji software using consistent settings. Before analysis, all images were rotated to properly align the anterior and posterior regions of embryos.

#### Analyses of endoderm cell orientation

*Z*-stack confocal images were acquired as described above and rotated to align with the AP and ML axes of the embryos. *XZ* images were extracted at positions determined by two criteria: (1) intact lateral endoderm cells; (2) clustering of lateral endoderm cells to form rosettes (using *z*-projection images as reference). In each *xz* image, lateral endoderm cells were outlined using the ‘Polygon’ tool and the outlines were saved using the region of interest (ROI) tool in Fiji software. The angles of the cells were determined using the ‘fit ellipse’ tool of Fiji software. The data were processed in Excel to determine the percentage of cells whose angles aligned within ±20° of the ML axis for each *xz* image. The average percentage across all images was calculated. The distribution of all angles from all cells in all embryos was plotted as rose diagrams using Past4 software.

#### Analyses of time-lapse movie

To determine defects in endodermal cells, we tracked the timelapse movies and quantified the number, area and duration of the gaps between endodermal cells. To determine the number and area of the gaps, snapshots of the movies were taken every 15 min. All gaps in the images were outlined using the ‘Polygon selection’ tool in Fiji software. The area of each gap was calculated, and the cell number in the image was determined. The average gap area and gap number per cell were determined. To evaluate the duration of gaps, we randomly tracked gaps in each embryo throughout the entire movie, excluding those that were not closed by the end of movie. The timing of gap closure was recorded, and the average time to close was determined for each gap. To determine the frequency of gap reopening, we tracked all gaps at every timepoint throughout the entire movie to identify which gaps reopened after closing. The percentage of gaps that reopened was calculated.

#### Analyses of the distribution of ZO-1 and myosin-GFP foci

To assess the localization of ZO-1 (Fig. 4A,B) and myosin-GFP foci (Fig. 5A,B) in pharyngeal endoderm at 10 ss, we analyzed *xz* plane images extracted from *z*-confocal stacks. The positions of ZO-1 and myosin-GFP foci on memGFP-labelled pharyngeal endoderm cells were manually examined. Multiple *xz* images were acquired from each embryo and independently examined by at least two individuals in a blinded fashion. The distribution of ZO-1 and myosin-GFP foci in different regions of endodermal cells (apical, basal and lateral) was determined by dividing the count in each category by the total foci number of ZO-1 and myosin-GFP in each *xz* plane. The average occurrence of each category in individual embryos was also calculated.

To evaluate the intensity and distribution of ZO-1 in pharyngeal endoderm cells at 20 ss (Figs 4E,F and 8A, Fig. S4A,B), we analyzed single *z*-plane *xy* images, focusing on the lateral region of the pharyngeal endoderm where the rosettes are situated. In images containing GFP-labelled endoderm and ZO-1 expression channels, narrow rectangular boxes (19×2 µm) spanning the rosette region were drawn and defined as a region of interest (ROI) using the rectangular tool in the ROI manager module. The position of the ROI boxes was adjusted to center the apical aspect of rosettes in the middle of rectangular boxes. Subsequently, GFP-labelled endoderm and ZO-1 expression channels were separated. ROI information was re-applied in the ZO-1-expressing images, and plot profiles were generated using the ‘multi plot’ function in the ROI manager module. The ZO-1 intensity (*y*-axis) across the endodermal rosette region (*x*-axis) was calculated. To evaluate the relative ZO-1 expression in embryos conducted on different dates (in Fig. 4E,F, Fig. S4A,B), the average ZO-1 intensity of each data point on the *x*-axis was calculated by taking the mean of the data from all control embryos conducted on the same date. The minimum average value was then identified. The relative ZO-1 intensity at each point across the endoderm in control and *gna13*-deficient embryos was determined by dividing the intensity at that point by the minimum average value from control embryos. For Fig. 8A, the ZO-1 intensity in donor cells was normalized to the intensity of the host cells in the same embryo. Subsequently, the average relative ZO-1 intensity over endodermal rosettes in all embryos was plotted.

To assess the localization of ZO-1 in the first endoderm pouch at 30 hpf (Fig. S5C,F), *xz* plane images were extracted from confocal *z*-stacks. The localization of ZO-1 on the endoderm cell membrane (labeled by mem-GFP) was analyzed. Rosette centers were defined as the apical region, whereas regions outside this area were classified as ectopic. The frequencies of ZO-1 localization in the apical and ectopic regions were determined in each *xz* plane. The average occurrence of each category was calculated for individual embryos.

#### Analyses of the distribution of pMLC

To assess the distribution of pMLC in the endoderm at 20 ss (Figs 5D,E and 8E,F), we analyzed *xz* plane images extracted from confocal *z*-stacks. The localization of pMLC expression on memGFP-labeled endoderm cells was examined. pMLC was expected to be present in apical rosette centers, whereas expression outside these centers was considered ectopic. The number of pMLC-expressing foci in different regions of endodermal cells (apical and ectopic) was determined, and their frequencies were calculated by dividing the count in each category by the total foci in each *xz* plane. Multiple *xz* images were acquired from each embryo and the number of pMLC-expressing foci was independently assessed by at least two individuals in a blinded fashion. The average occurrence of each category in individual embryos was calculated.

#### Analyses of the expression E-cadherin and N-cadherin

To assess E-cadherin expression in the pharyngeal endoderm, we examined single *z*-plane *xy* images at the 12 ss and 20 ss stages. At 12 ss, E-cadherin expression was concentrated on the endodermal plasma membrane with minimal presence in the cytosol. Narrow rectangular boxes were delineated to exclusively encompass the plasmas membrane of *sox17*:memGFP*-*expressing endodermal cells, and these were defined as ROIs using the rotated rectangular tool in the ROI manager module. At 20 ss, E-cadherin was observed in both the plasma membrane and the cytosol of endodermal cells. Consequently, rectangular ROI boxes that were each 3 µm in height and spanned the rosette region of the endoderm were delineated. Subsequently, E-cadherin-expressing images were converted into 32-bit images and merged with ROI information acquired from the GFP channel. The same values of ‘lower threshold’ and ‘upper threshold’ were applied to all images with the background set to NaN. The mean gray value and the area value (µm^2^) within the specified threshold ranges were measured. These two values were then multiplied to obtain the total intensity, which was then divided by the area (µm^2^) of the ROI to derive the intensity of the ROI (I_ROI_). Finally, the average I_ROI_ in control embryos was used to evaluate the relative E-cadherin expression in *gna13*-deficient embryos.

### Statistical analysis

Data were gathered from two to three independent experiments and expressed as the mean±s.e.m. Statistical analyses were conducted in GraphPad Prism (GraphPad Software) using unpaired two-tailed Student's *t*-tests with unequal variance, one-way ANOVA analyses and the chi-square test, as indicated in figure legends. Significance was set at *P*<0.05. Various symbols were employed in the figures to denote the different *P* values: n.s., *P*>0.05; *P<0.05; **P<0.01; ***P<0.001; ****P<0.0001. The number of cells and embryos analyzed in each experiment is indicated in the figure legends.

## Supplementary Material



10.1242/develop.202597_sup1 Supplementary information
