# Supplementary material for: Gα13 controls pharyngeal endoderm convergence by regulating E-cadherin expression and RhoA activation
Source: Development. 2024 Sep 30;151(19):dev202597. doi: 10.1242/dev.202597 (PMC11463957; doi:10.1242/dev.202597)
Supplement: Supplementary information [file develop-151-202597-s1.pdf]

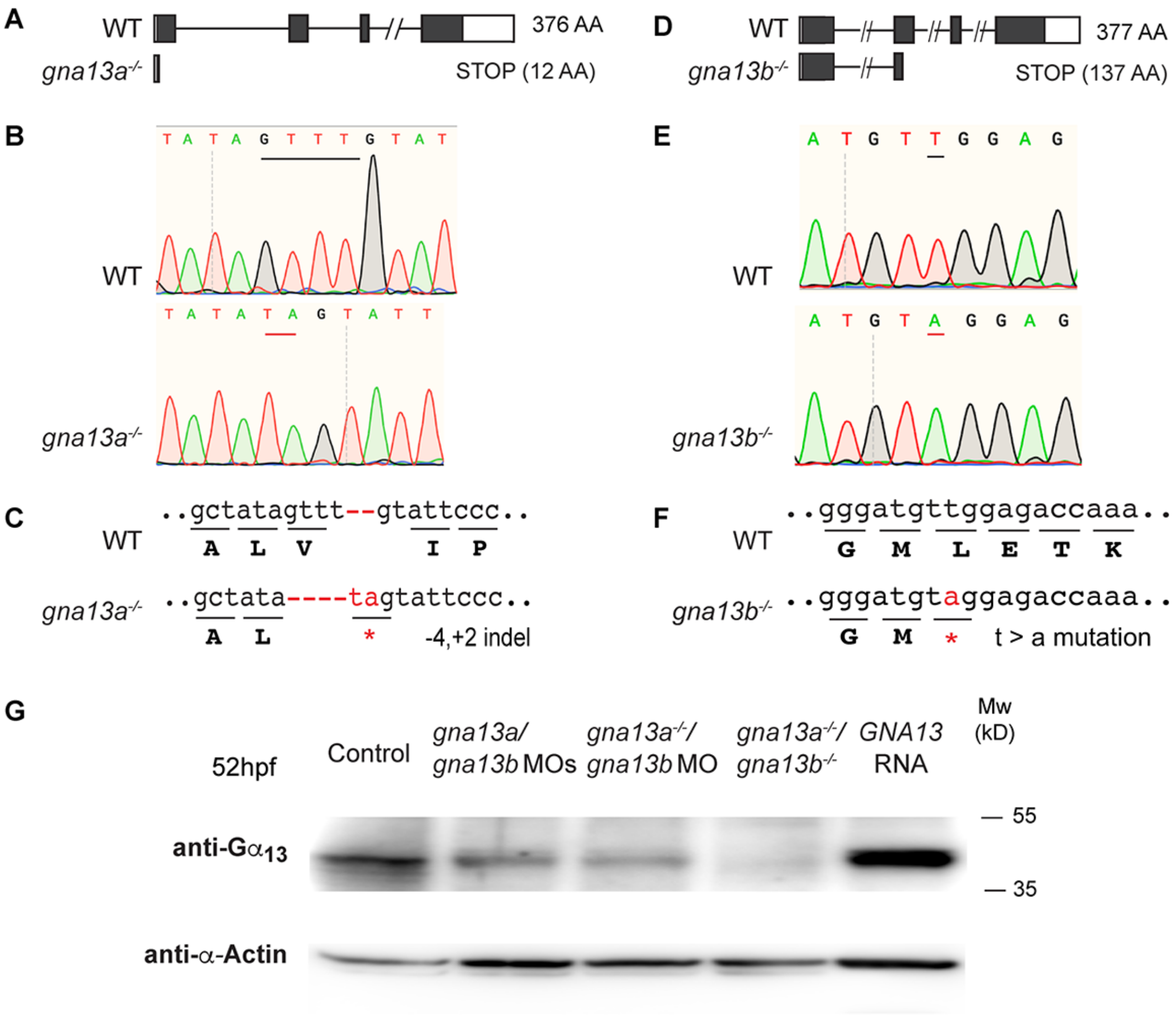


Figure S1

**Fig. S1. Generation of *gna13a* and *gna13b* mutant zebrafish.**

(**A-C**) Genomic characterization of *gna13a*$^{-/-}$ mutants. (A) Schematic showing the wild-type (WT) allele (full length is 376 AAs), and the *gna13a* mutant allele (only 12 AAs). Black box, exons; White box, UTR; Lines: introns. (B) Chromatograms of genomic DNA sequences showing mutations in the *gna13a*$^{-/-}$ allele in exon 1. Four base pairs (bp) in the WT allele were deleted (black line) and 2 bp were inserted (red line). (C) A stop codon (red asterisk) was created in the *gna13a*$^{-/-}$ allele. (**D-F**) Characterization of

*gna13b*$^{-/-}$ mutants. (D) Schematic showing the WT allele (full length is 377 AAs) and the *gna13b* mutant allele (137 AAs). (E) Chromatograms of genomic DNA sequences showing a mutation in the *gna13b*$^{-/-}$ allele in exon 2. “T” (black underline) is replaced with “A” (red underline). (F) A stop codon (red asterisk) was created in the *gna13b*$^{-/-}$ allele. (**G**) Western blot of $G\alpha_{13}$ and $\alpha$-Actin (a loading control) expression in the indicated embryos at 52hpf.

Development • Supplementary information

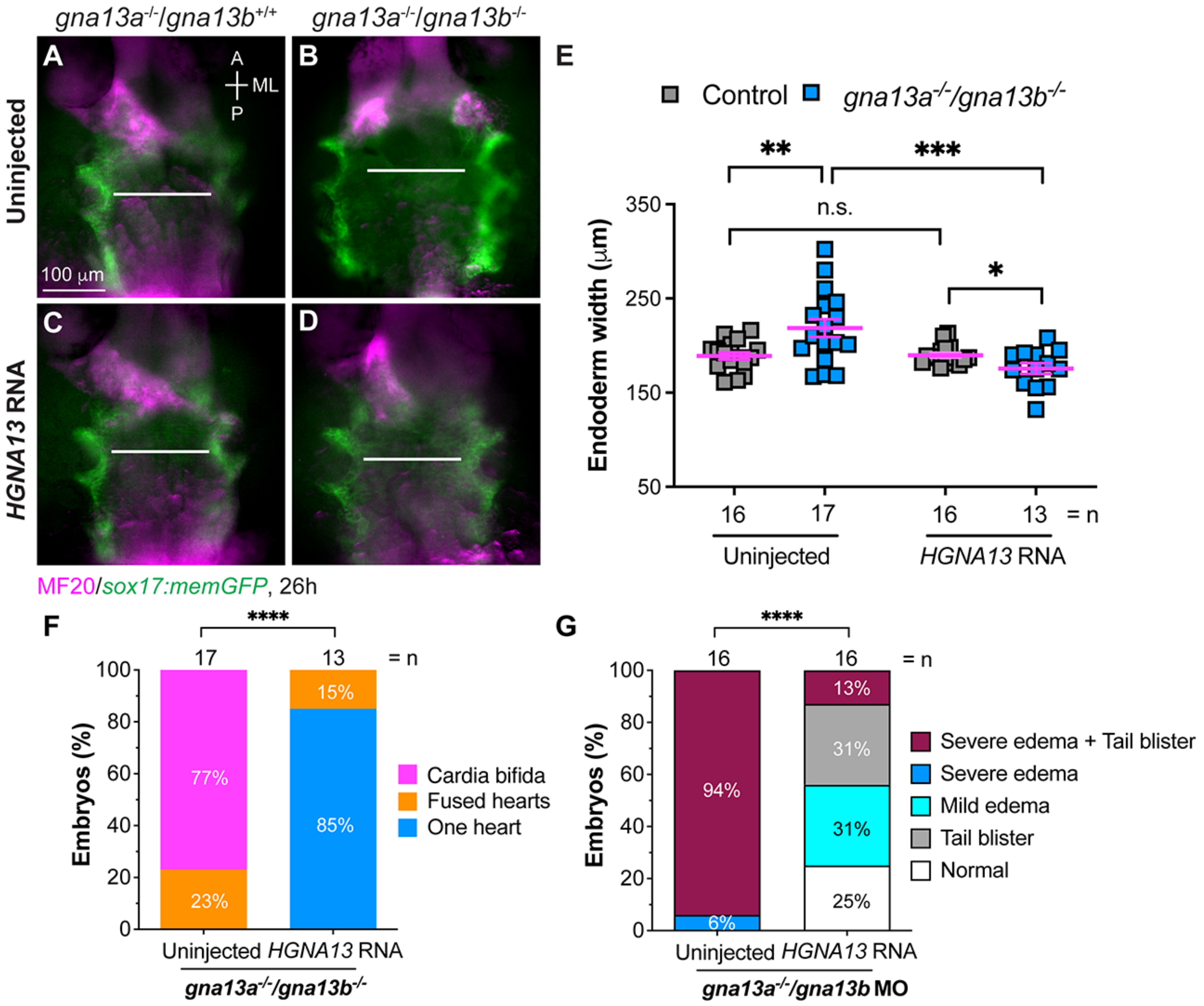


Figure S2

**Fig. S2. Expressing human G$\alpha_{13}$ rescues the defects in *gna13a/gna13b*- deficient embryos.**

(**A-D)** Epifluorescence images of the pharyngeal endoderm at 26hpf in the indicated embryos showing *sox17:memGFP*-labeled pharyngeal endoderm and the locations of cardiomyocytes detected by MF20 immunostaining. Dorsoanterior view with anterior upwards. White lines of equivalent length, the width of the pharyngeal endoderm sheet. A, Anterior; P, Posterior; ML, Mediolateral. (**E**) Average width of the pharyngeal

Development • Supplementary information

endoderm in indicated embryos shown in A-D. The number of embryos analyzed in each group is indicated. Data are mean±s.e.m. n.s. (not significant), $P>0.05$; *$P<0.05$; **$P<0.01$; ***$P<0.001$, unpaired, two-tailed Student's *t*-test. (**F)** The percentage of embryos with various classes of heart morphology phenotypes [normal heart, two fused hearts, and two separated hearts (cardia bifida)] in the indicated embryos. The total number of embryos is indicated. ****$P<0.0001$, chi-square test. (**G**) The percentage of phenotypic classes of cardiac edema and tail blistering (as described in Fig. 1) in the indicated embryos at 49hpf. The total number of embryos is indicated. ****$P<0.0001$, chi-square test.

Development • Supplementary information

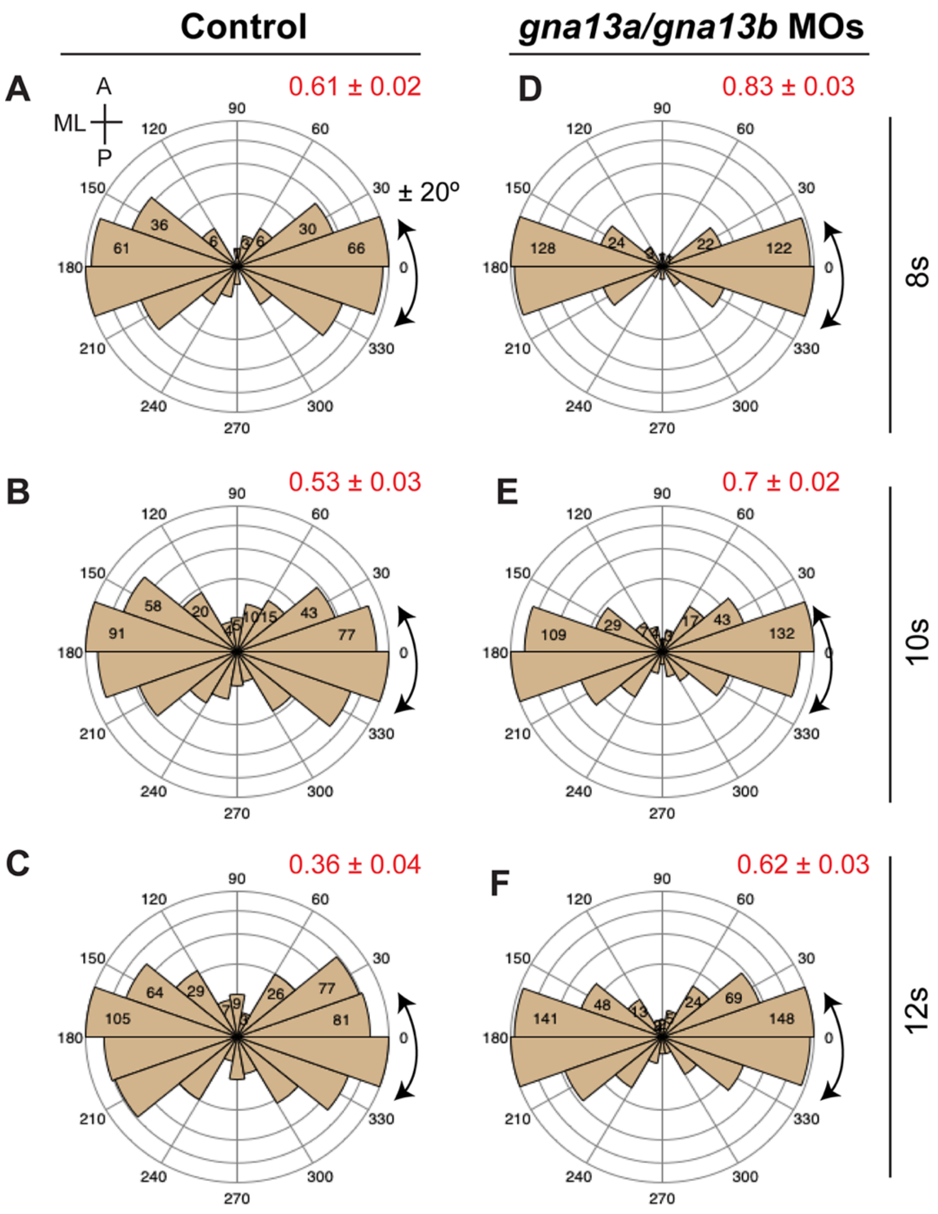


Figure S3

**Fig. S3. Gα13 regulates the orientation of endodermal cells during endoderm C&E.**

Rose plots illustrate the distribution of endoderm cells' orientation in control (A-C) and *gna13a*/*gna13b* MOs-injected (D-F) embryos at the indicated stages, which shown in Figure 2. Each bin represents 20°. The numbers indicate the average percentage of ML-aligned (±20°) endodermal cells.

Development • Supplementary information

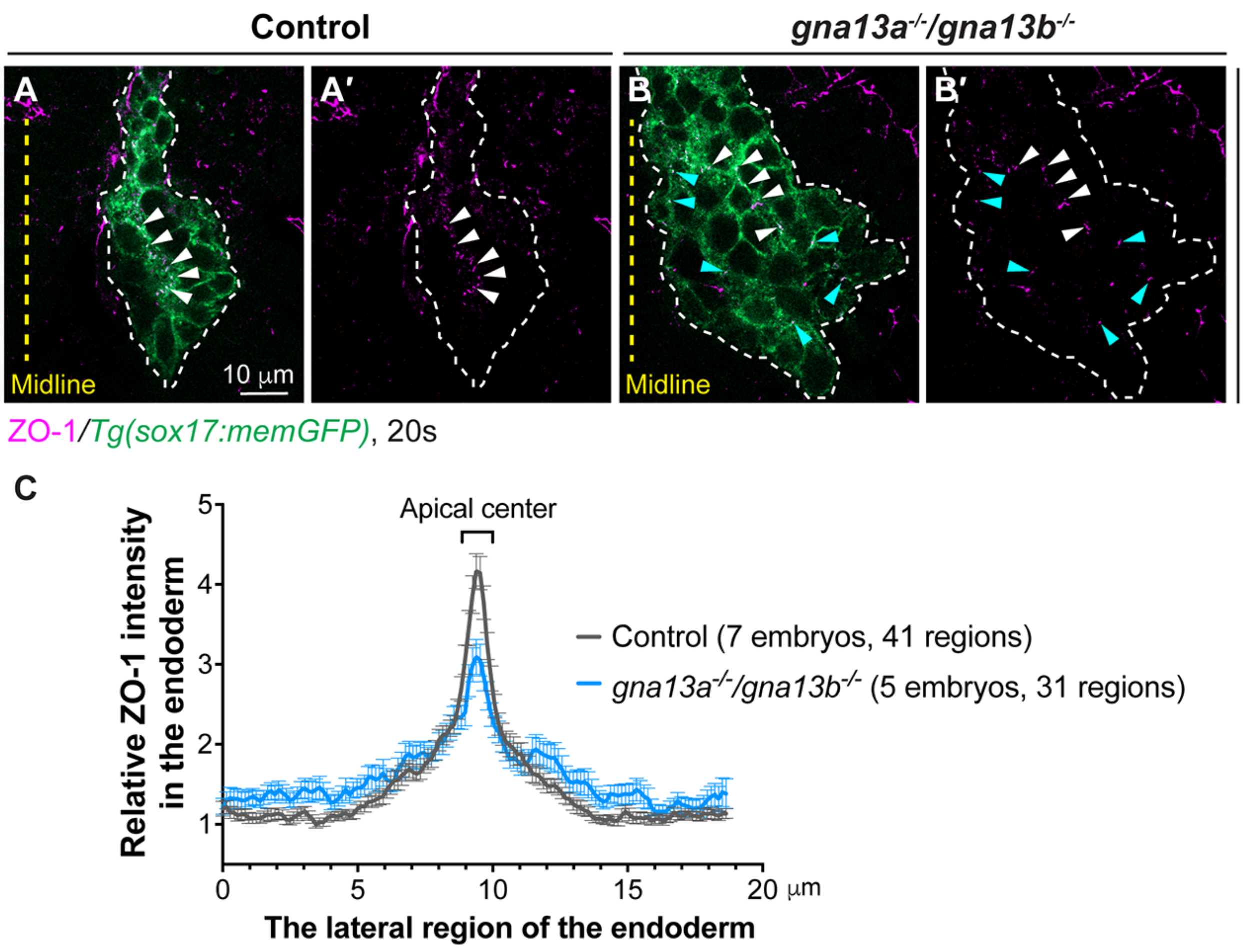


Figure S4

**Fig. S4.** ***gna13a$^{-/-}$/gna13b$^{-/-}$*** **embryos exhibit defects in the apical constriction of endodermal cells.**

Whole-mount immunostaining was conducted to assess ZO-1 localization in offspring obtained from incrossing *gna13a$^{-/-}$/gna13b$^{+/-}$* fish. Confocal imaging was performed, and the genotype of each embryo was determined post-imaging. (**A-B'**) Images of a single confocal Z-plane in XY view displaying ZO-1 localization (magenta) in endodermal cells (green, labelled by memGFP) in the indicated embryos at 20s. White arrowheads, ZO-1 puncta in the apical region; Cyan arrowheads, ZO-1 puncta in the ectopic regions. White dashed lines, outline of the endoderm. Yellow dashed line, midline. (**C**) Average relative intensity of ZO-1 expression across the lateral region of the endoderm in XY planes. The number of regions of images and embryos is indicated.

Development • Supplementary information

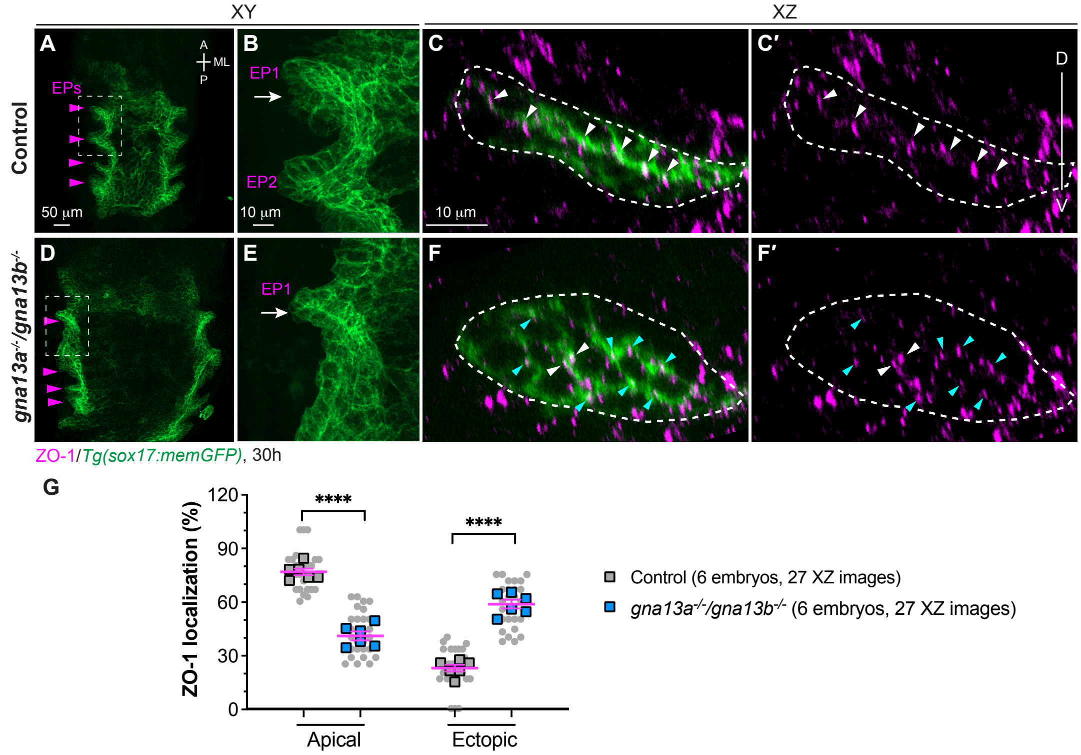


**Fig. S5. *gna13a$^{-/-}$/gna13b$^{-/-}$* embryos exhibit defects in the endoderm pouch.** Whole-mount immunostaining of ZO-1 localization in the indicated embryos at 30 hpf. (**A,B,D,E**) Confocal Z projection images showing the morphology of pharyngeal endoderm. Boxes with dashed lines, areas where high magnification confocal images (B,E) were taken. Magenta arrowheads, endoderm pouches (EPs). A, anterior; P, posterior; ML, mediolateral. (**C,F,G**) ZO-1 localization (magenta) in pharyngeal endodermal cells (green, labelled by memGFP) in the indicated embryos. (C',F') Images of XZ planes, taken at positions marked by white arrows in C and F. White dashed lines outline the endoderm. Arrowheads, ZO-1-expressing puncta in the apical (white arrowheads) and ectopic (cyan arrowheads) regions; D, Dorsal; V, Ventral. (G) The frequencies of ZO-1-labeled puncta in the apical and ectopic regions in the indicated embryos. Data from all embryos (squares) and all XZ images (gray circles) are superimposed, with the number of XZ images and embryos indicated. Data are mean±s.e.m. ****$P$<0.0001 (unpaired, two-tailed Student's $t$-test).

Development • Supplementary information

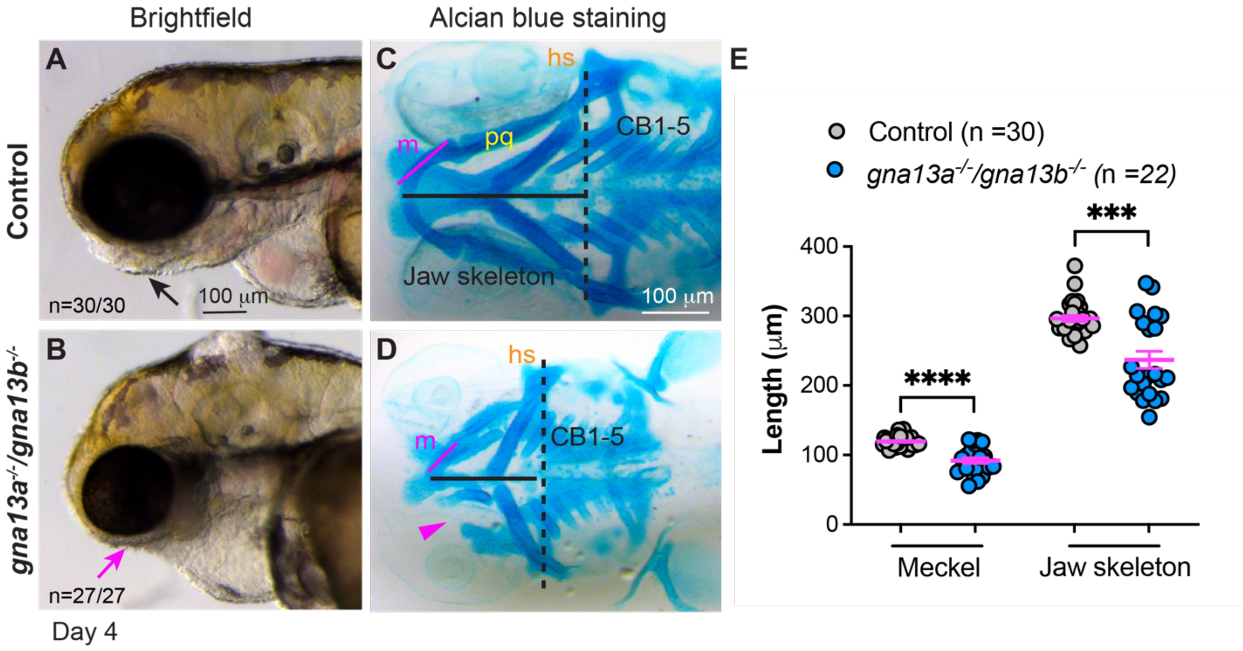


Figure S6

**Fig. S6. *gna13a$^{-/-}$/gna13b$^{-/-}$* embryos exhibit defects in jaw development.**

(**A,B**) Brightfield images of control and *gna13a$^{-/-}$/gna13b$^{-/-}$* embryos at day 4. Lateral view. Black arrow, jaw in control embryo (30 embryos); magenta arrow, jaw in *gna13a$^{-/-}$ /gna13b$^{-/-}$* embryos (all 27 embryos had similar defects). (**C,D**) Whole-mount images of dissected facial cartilage detected by Alcian Blue staining. Ventral view. m, Meckel's cartilage (magenta line); pq, palatoquadrate cartilage; hs, hyosymplectic cartilage; Magenta arrowhead, the missing Meckel's cartilage in one side of the mutant embryo; Black line, the length of the jaw skeleton (the distance from the anterior end of Meckel's cartilage to the hyosymplectic cartilage). (**E**) The average length of Meckel's cartilage and the jaw in the indicated embryos. The number of embryos analyzed in each group is indicated. Data are mean±s.e.m. ***$P$<0.001, ****$P$<0.0001 (unpaired, two-tailed Student's $t$-test).

Development • Supplementary information

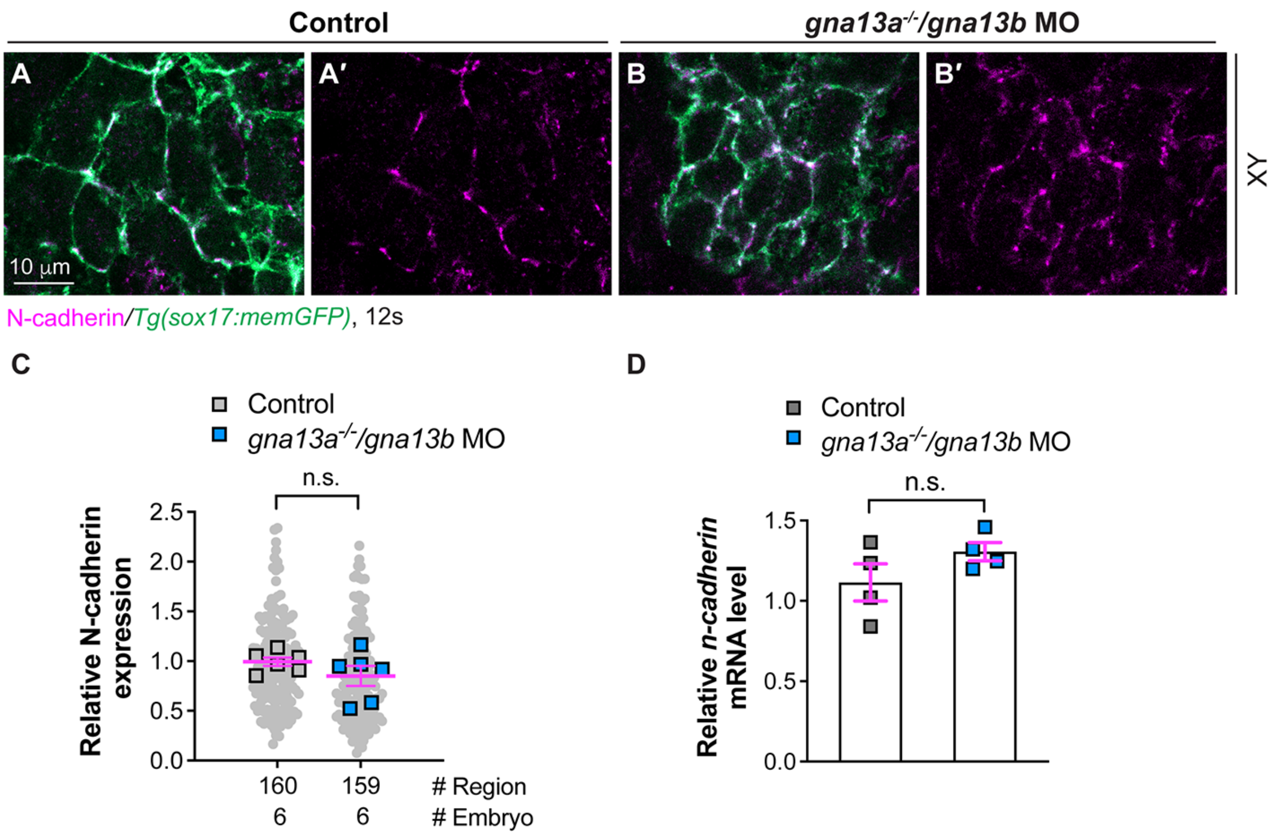


Figure S7

**Fig. S7. The expression of N-cadherin is not altered in *gna13a/gna13b*-deficient embryos.**

(**A-C)** The localization of N-cadherin detected by whole mount immunostaining. (A-B') Single confocal Z plane images in indicated embryos at 12s. (C) Relative intensity of N-cadherin expression on the plasma membrane of endodermal cells in the indicated embryos shown in A,B. Data from all embryos (squares) and regions (gray circles) are superimposed, with the number of regions and embryos indicated. (**D**) qPCR of *cdh2* mRNA in the indicated embryos at 9s. Four experiments were performed. Data are mean±s.e.m. n.s. (not significant), *P*>0.05, (unpaired, two-tailed Student's t-test).

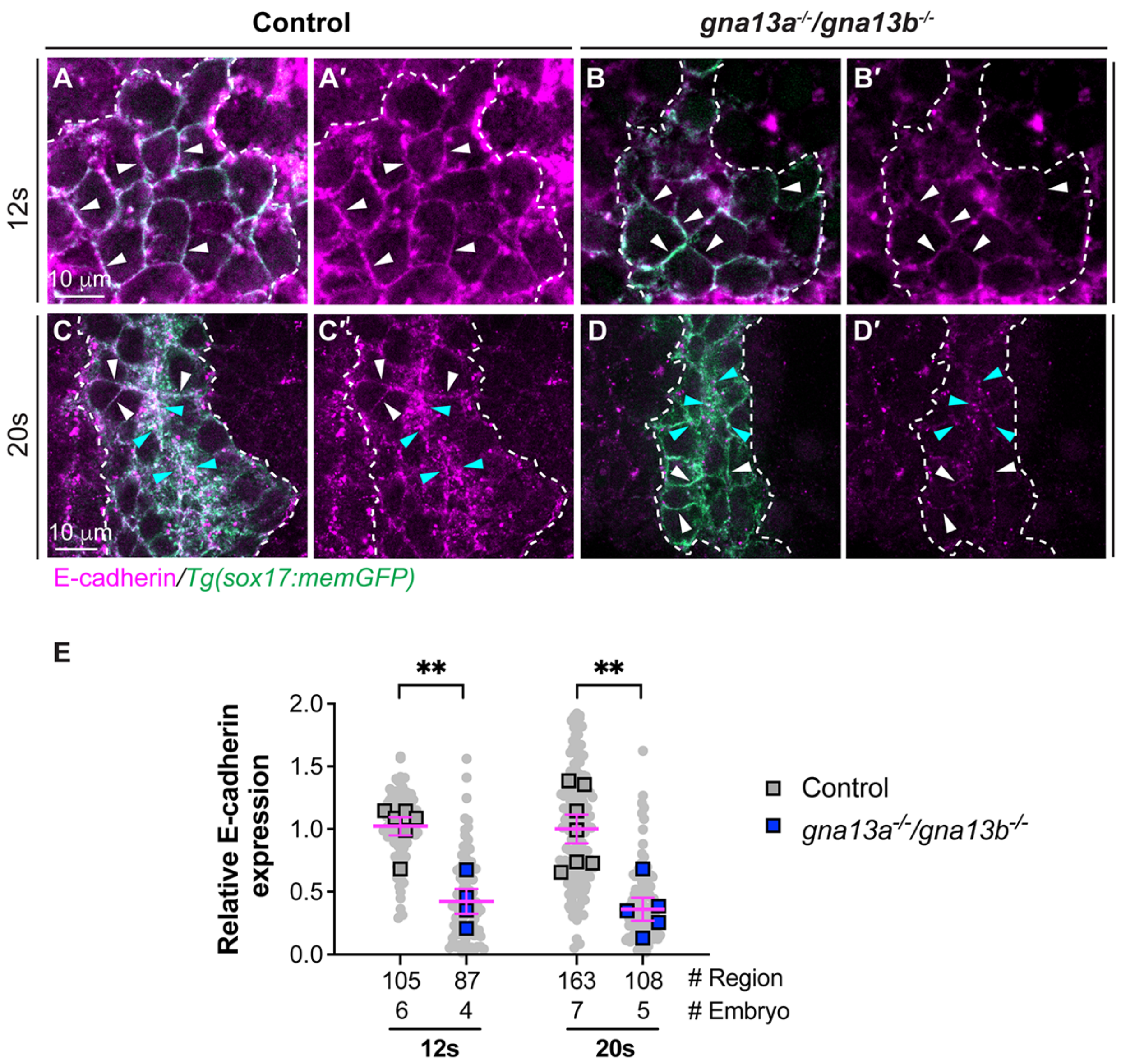


Figure S8

**Fig. S8. E-cadherin abundance is reduced in *gna13a$^{-/-}$/gna13b$^{-/-}$* embryos.**

Whole-mount immunostaining for E-cadherin localization (magenta) in endodermal cells (green) in the indicated embryos at 12s and 20s. (**A-D'**) Single confocal Z plane in the XY view. White arrowheads, E-cadherin localization on plasma membrane of endoderm cells; Cyan arrowheads, E-cadherin-enriched puncta in the cytosol of endodermal cells; Dashed lines, the endoderm boundary. (**E**) Relative intensity of E-cadherin staining on

Development • Supplementary information

the plasma membrane of endodermal cells at 12s in A, B and the regions in the endodermal rosettes at 20s in C, D, in the indicated embryos. Data from all embryos (squares) and regions (gray circles) are superimposed, with the number of regions and embryos indicated. Data are mean±s.e.m. ***P*<0.01 (unpaired, two-tailed Student's t-test).

Development • Supplementary information

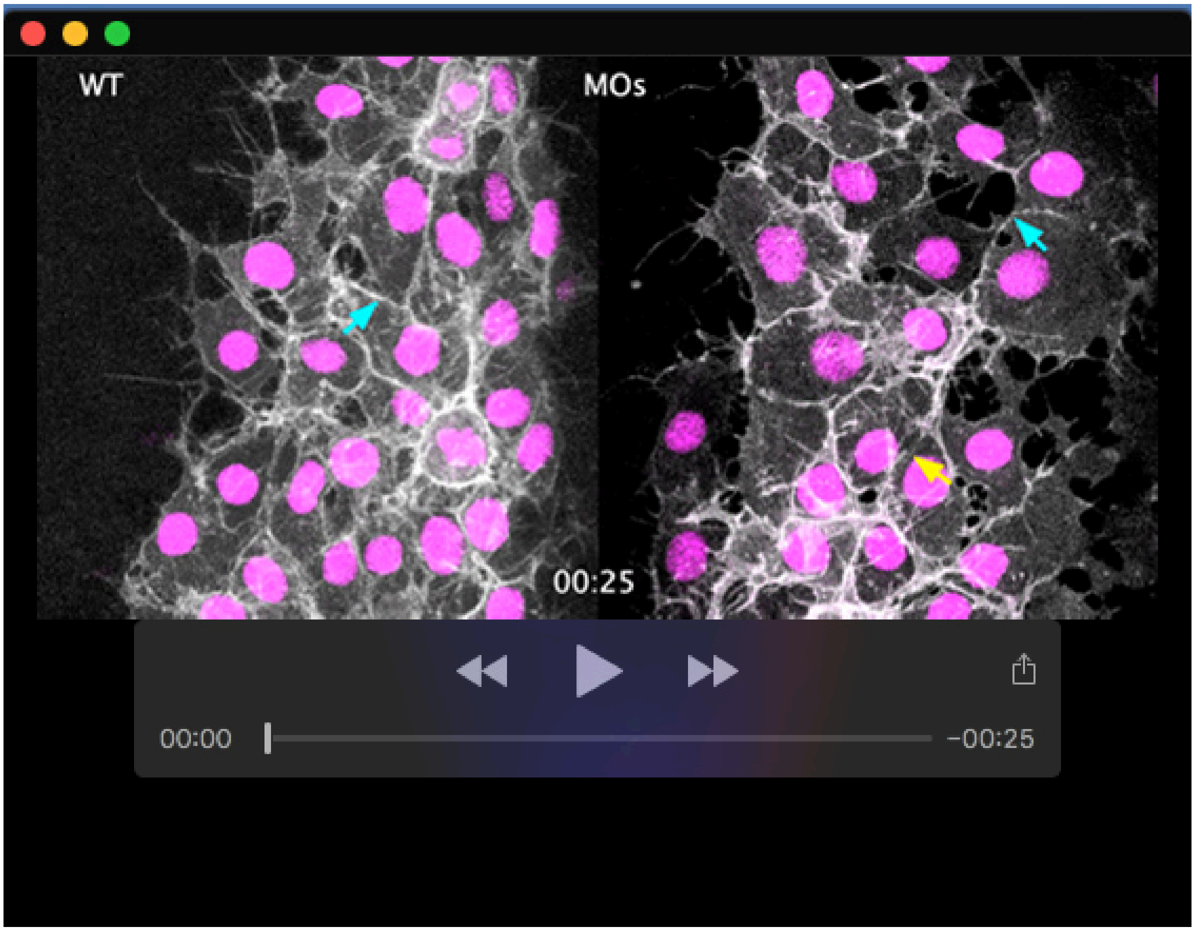


**Movie 1. G$\alpha_{13}$ regulates stable cell-cell contacts during pharyngeal endoderm C&E.** Confocal time-lapse experiments were performed on *Tg(sox17:memGFP/H$_2$A-mCherry)* control (WT) and *gna13a/gna13b* MOs-injected (MOs) embryos at 7-9 somite stages using a Zeiss LSM880 confocal microscope with an LD C-Apo 40×/NA 1.1 water objective. Z-stacks of 16.5 μm were acquired at 1.5 μm intervals every 5 minutes, employing the following settings: zoom 1.2, 1024X1024 pixels, 7 speed, 2 averaging. The movie plays at 3 frames per second. Arrows of the same color indicate the same gap over time, with some gaps closing at specific time points.

Development • Supplementary information

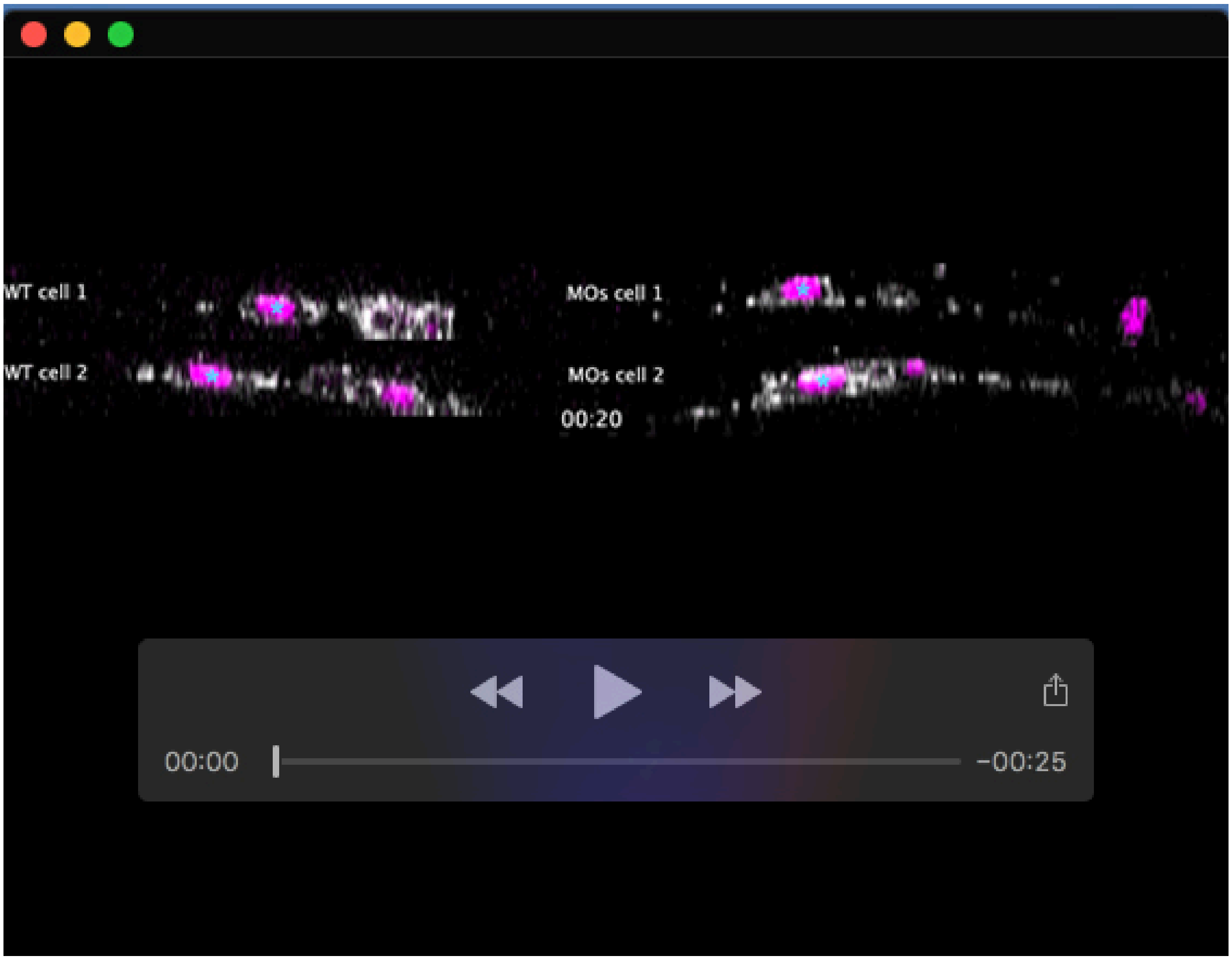


**Movie 2. $G\alpha_{13}$ is required for the orientation changes of endoderm cells during C&E.**

The movie displays images of the XZ plane, extracted from confocal z-stacks used in Movie 1, every 10 minutes. Two cells from control (WT) or *gna13a/gna13b* MOs-injected (MOs) embryos were tracked, and their nuclei were marked by cyan asterisks. The movie plays at 2 frames per second, illustrating the orientation changes of the nuclei of two cells at the indicated time points.

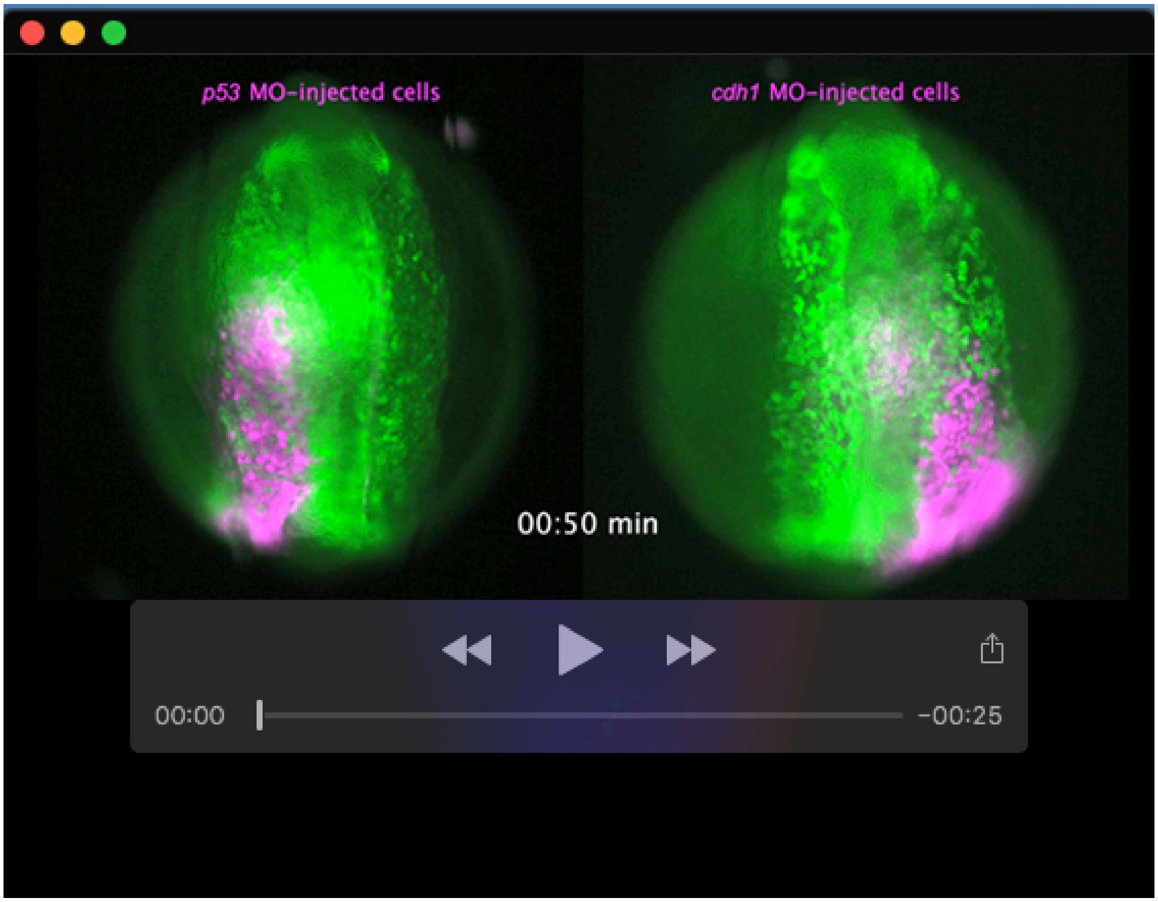


**Movie 3. E-cadherin is required for efficient pharyngeal endoderm C&E during early segmentation.**

Representative time-lapse movie of pharyngeal endoderm of *Tg(sox17:EGFP)* embryos transplanted with indicated cells (magenta), from 7-10s. Images were captured at 5-min intervals using an epifluorescence microscope (DMI 6000, Leica) with a 5x/NA 0.15 objective. The movie plays at 5 frames per second.

Development • Supplementary information
