# Supplementary material for: Gα13 controls pharyngeal endoderm convergence by regulating E-cadherin expression and RhoA activation
Source: Development. 2024 Sep 30;151(19):dev202597. doi: 10.1242/dev.202597 (PMC11463957; doi:10.1242/dev.202597)
Supplement: Supplementary file 1 [file develop-151-202597_review_history.pdf]

$G\alpha_{13}$ controls pharyngeal endoderm convergence by regulating E-cadherin expression and RhoA activation

Bo Hu, Joshua Pinzour, Asmi Patel, Faith Rooney, Amie Zerwic, Yuanyuan Gao, Nhan T. Nguyen, Huaping Xie, Ding Ye and Fang Lin
DOI: 10.1242/dev.202597

Editor: Steve Wilson

Review timeline

Original submission:	8 December 2023
Editorial decision:	22 January 2024
First revision received:	15 July 2024
Editorial decision:	19 August 2024
Second revision received:	19 August 2024
Accepted:	28 August 2024

Original submission

First decision letter

MS ID#: DEVELOP/2023/202597

MS TITLE: Gα13 controls pharyngeal endoderm convergence by regulating E-cadherin expression and RhoA activation

AUTHORS: bo hu, Joshua Pinzour, Asmi Patel, Faith Rooney, Amie Zerwic, Yuanyuan Gao, Nhan T Nguyen, Huaping Xie, Ding Ye, and Fang Lin

I have now received all the referees' reports on the above manuscript, and have reached a decision. The referees' comments are appended below, or you can access them online: please go to BenchPress and click on the 'Manuscripts with Decisions' queue in the Author Area.

As you will see, the referees express considerable interest in your work, but have some suggestions to ensure your data/conclusions are robust and to improve other aspects of thestudy. If you are able to revise the manuscript along the lines suggested, I will be happy receive a revised version of the manuscript. Please also note that Development will normally permit only one round of major revision. If it would be helpful, you are welcome to contact us to discuss your revision in greater detail. Please send us a point-by-point response indicating your plans for addressing the refereesâ€™ comments, and we will look over this and provide further guidance.

Please attend to all of the reviewers' comments and ensure that you clearly highlight all changes made in the revised manuscript. Please avoid using 'Tracked changes' in Word files as these are lost in PDF conversion. I should be grateful if you would also provide a point-by-point response detailing how you have dealt with the points raised by the reviewers in the 'Response to Reviewers' box. If you do not agree with any of their criticisms or suggestions please explain clearly why this is so.

Reviewer 1

Advance summary and potential significance to field

© 2024. Published by The Company of Biologists under the terms of the Creative Commons Attribution License (https://creativecommons.org/licenses/by/4.0/).

This study investigates a role for G alpha 13 in endoderm development in zebrafish. It makes good use of genetic mutants and knockdowns, impressive time-lapse imaging, transgenic and antibody labeling of apicobasal polarity markers and mosaic analysis. While endoderm phenotypes have previously been reported for morpholino knockdown of gna13a/b, a strength of this paper is validation of phenotypes using genetic gna13a/b mutants. This should be moved to the main figure rather than relegated to Fig. S4. Based on similar phenotypes of knockdown and genetic mutant embryos, it is reasonable to use the mutant/knockdown combination for several of the experiments though further validation with genetic mutants would increase rigor of study (in particular E-cadherin reduction Â– this should be performed on mutant clutches and scored before genotyping to reduce bias). I also did not find the p-MLC changes in Fig. 6 and S5 to be particularly convincing, and these lacked any quantitative assessment, sample size, or statistics. It was also unclear why supplying E-cadherin RNA rescued gna13a/b embryos if E-cadherin is regulated at the protein and not transcriptional level. If these issues can be addressed, I think this is an impactful study of the cell biological processes controlled by G alpha 13 and how defects in these affect endodermal adhesion midline migration, and subsequent pouch formation required for craniofacial development.

Comments for the author

1. Not clear what arrowheads are pointing to in Fig. 1I. Its also unclear what is being referred to as “enriched expression” as earlier stages in MOs do not look much different (8s and 10s).

2. Data for MOs in Fig 1F’-J’ are not compelling. How consistent are these phenotypes? This needs quantitation across multiple animals to be convincing.

3. The green label in Fig. 3 should be stated in legend

4. Lines 314-315, the second (not first) PA is situated between the first and second EP

5. Fig S5E,J - images of pMLC in controls vs. MOs are not convincing. How consistent are these defects? Needs quantification across multiple embryos.

6. Transgenic analysis of myosin-GFP is a strength - Fig S5 should be made a main figure

7. Fig 4J - strong reduction of E-cad protein in whole embryo at 9s is a bit surprising as presumably E-cad is also expressed in many non-endodermal epithelial cells at this stage. What is the explanation for this?

8. N-cadherin data mentioned on p. 17 should be shown

9. Lines 382-383. Should say “Fig 4K”. If E-cad levels are regulated at the protein and not transcriptional level, why does providing more mRNA rescue the defect? While rescue of gna13 knockdown by ecad RNA in Fig 5G-J is a nice result this is again confusing if E-cad levels are controlled at the protein and not mRNA level.

10. In the clonal E-cad MO experiments of Fig. 6, the reduction of ZO1 is convincing but not the reduction of p-MLC. Again, how consistent are these defects? Needs quantification across multiple embryos.

11. Legend to Fig 7F,G - should say white arrows (not arrowheads)

12. In Methods, it is stated that mem-GFP RNA was used as lineage tracer for endoderm transplants but I don’t see this experiment in any figure.

13. Quantification (at minimum number of embryos examined and how often the shown phenotype was observed) needs to be provided for each experiment. For example Fig. S4 but many others.

<u>Reviewer 2</u>

© 2024. Published by The Company of Biologists under the terms of the Creative Commons Attribution License (https://creativecommons.org/licenses/by/4.0/).

Advance summary and potential significance to field

In this study, Hu et al. investigate mechanisms controlling early morphogenesis of the pharyngeal endoderm in zebrafish embryos, preceding the formation of endodermal pouches. This group previously reported roles for Ga13a and Ga13b in pharyngeal endoderm convergence, myocardial cell migration, and caudal fin formation, using morpholino knockdowns (Ye and Lin, 2013). Here they probe deeper using genetic loss of function models, finding that deficiency of Ga13 proteins interferes with medial convergence, rosette formation, and cell-cell adherence of the pharyngeal endoderm sheet, though not formation of endodermal pouches. Using a clever set of transplant and rescue experiments, they tie convergence phenotypes to reduced levels of E-cadherin protein and disruption of cytoskeletal changes associated with apical constriction. Some but not all of these processes appear to be mediated by RhoA downstream of Ga13a.

Beyond the Ga13a studies, the authors also provide a wealth of data on the basic biology of the endodermal convergence process, which will be of value for the field. Overall, this well-controlled, carefully-quantified (with an exception detailed below) study investigates an essential early step in endodermal development that organizes the tissue for subsequent organ development.

Comments for the author

The main weakness is the lack of quantification provided for the morphogenetic events presented in Figs. 1 and 2. As is, it is unclear how consistent these patterns are between embryos of the same genotype/stage.
e.g. How commonly do the “holes” in the pharyngeal sheet appear? Are the changes in nuclear orientation reproducible?

Minor comments:

Line 43: Change “the innermost layers of the germ layer” to “the innermost of the germ layers”

Lines 58-62: The repeated use of the word “specific” in this section is problematic, as the authors haven’t given enough detail for the reader to understand what makes the pharyngeal endodermal sheet “specific” or possessing a “specific architecture”, or what is “specific” about the points where pouches emerge.

Line 70: The authors write that “pharyngeal endoderm cells do not exhibit polarity”, in the context of non-canonical Wnt signaling. If they are referring only to planar cell polarity, they should make that explicit; otherwise it is confusing, as this paper describes a role for Ga13 in establishing apicobasal polarity.

Lines 131-132: There is a missing adjective after “exhibited” in the sentence: “Additionally, gna13a-/-/gna13b-/- embryos frequently exhibited heads and eyes”. Reduced? Abnormal?

Line 314: The statement that “The first PA is situated between the first and second EP” is incorrect – the first endodermal pouch separates the first and second pharyngeal arches. The observed defects in Meckel’s cartilage (derived from the first pharyngeal arch) may be attributable not to pouch defects but rather to abnormalities in the more anterior endodermal cells that contact the oral ectoderm.

Fig. 3: Provide labels for the stains/reporters used in this figure.

Lines 411-412: Please use the gene name for E-cadherin (cdh1) when referring to the RNA injections.

Lines 432-434: Please provide more detail in the text on this experiment involving H2B-GFP RNA injections – was this the same protocol as shown in Fig. 5a. (i.e. endoderm transplants), but with this nuclear-targeted GFP used as a marker instead of Rhodamine-dextran?

Line 863: Replace “chd1” with “cdh1”

© 2024. Published by The Company of Biologists under the terms of the Creative Commons Attribution License (https://creativecommons.org/licenses/by/4.0/).

Reviewer 3

Advance summary and potential significance to field

In this manuscript, Hu and colleagues examine the molecular mechanisms that control pharyngeal endoderm convergence and extension in the zebrafish embryo. Through elegantly performed live imaging at the cellular and subcellular level, together with time-lapse and mosaic analysis, they implicate two pathways downstream of G-alpha13 in the control of pharyngeal endoderm cell behavior. One of these pathways activates RhoA and controls actomyosin dynamics, and the other pathway controls localization of E-cadherin, with consequences both for cell-cell adhesion and actomyosin dynamics. The careful work performed here is high-quality, and it highlights the potential for context-specific differences in the roles played by G-alpha13. In this regard, it will be of interest to developmental biologists interested in the cellular and molecular mechanisms of tissue morphogenesis in a variety of contexts. However, certain aspects of the manuscript would benefit from clarification or elaboration. Additionally, some of the most interesting open questions raised by the results are not addressed here, although these are probably considered to be beyond the scope of the current study.

Comments for the author

Major points:

- In Figures S1, S2, and S3, the authors compare some aspects of the gna13a;gna13b double homozygotes with other genotypes, with gna13a homozygotes injected with a gna13b MO, and with embryos injected with both the gna13a and gna13b MOs. These comparisons are intended to demonstrate that the gna13a;gna13b double homozygotes resemble the gna13a homozygotes injected with a gna13b MO and the embryos injected with both the gna13a and gna13b MOs, so that subsequent experiments can take advantage of the convenience of working with the MOs and do not need to use the double homozygotes.
It's understandable why the authors would take this approach, but it is important to confirm that the key phenotypic observations, particularly those made in Figures 3 and 4 (regarding ZO-1 localization and E-cadherin levels and localization) are also apparent in the gna13a;gna13b double homozygotes. Hopefully the authors now have the appropriate fish carrying both mutations and the sox17:memGFP transgene so that these experiments can be performed during the standard revision timeframe.

- In Figure 1, it would be helpful to clarify whether the midline is on the left side of all of the XZ sections shown. The orientation is unclear, as it seems that some of these sections are taken from the left side of the pharyngeal endoderm whereas others are taken from the right side of the pharyngeal endoderm.
Some of the descriptions of phenotypes displayed in Figure 1, particularly the descriptions of cell shape and orientation, are not easy to discern from the the images shown. It is certainly clear that rosette formation and tissue thickening do not proceed normally in the MO-injected embryos, but the other observations would benefit from quantification or clarification. The authors should consider whether to modify the text to be more compatible with the qualitative nature of Figure 1 or whether to add quantification to their observations.

- Similarly, in Figure 2, while the effect of the MOs on cell-cell contacts is easy to discern, the described differences in cell shapes and orientations are not as clear in the figure, and quantification and/or clarification would help. For example, for the nuclei that are tilting ventrally near the end of the wild-type time-lapse, is the whole cell (and the whole tissue) tilting or just the nucleus?

- In Figure S5, the contrast between organized and disorganized myosin-GFP localization is clear, but the relative concentration on the apical surface is less evident in the images. Quantification of this localization, similar to that performed for ZO-1 in Figure 3, would be valuable.

© 2024. Published by The Company of Biologists under the terms of the Creative Commons Attribution License (https://creativecommons.org/licenses/by/4.0/).

- The results shown in Figure 5G-K don't seem to make sense — if E-cadherin transcript levels are normal in the gna13-deficient embryos, then why does injection of cdh1 RNA rescue endoderm width? How do the authors reconcile this?

- As the authors point out in their discussion, their studies do not resolve how G-alpha13 regulates the spatial activation of actomyosin or the levels and localization of E-cadherin. These are not easy questions to answer, and they likely fall beyond the scope of the current manuscript, but these are certainly the ways that this line of investigation could have the largest impact on our understanding of the molecular mechanisms regulating convergent extension.

Minor points:

- While the manuscript is generally clearly written, it would be beneficial for the authors to proofread carefully for typos, examples of which include: "Wnt111r" should be "Wnt11r", "alternations" should be "alterations", "titled" should be "tilted", etc. Also, some language can be simplified: for example, it is not necessary to say "genetic mutant" or "genetic mutations", since "mutant" and "mutations" are sufficient to convey the appropriate meaning.

- Perhaps this view is too old-fashioned, but it would be preferable to discuss ZO-1 "localization" rather than "expression" when describing the results in Figure 3, reserving "expression" for the description of transcriptional regulation and "localization", "levels", "presence", etc., to describe where proteins are found. Similarly, it would be helpful to change the semantics to discuss "localization", "levels", "presence", etc., of E-cadherin protein and "expression" of E-cadherin transcripts.

First revision

Author response to reviewers' comments

Overall response:

We appreciate the reviewers' careful consideration and overall positive reception of our manuscript. We found their constructive comments and suggestions helpful in revising our study. We have carefully addressed all issues that were raised, as outlined in detail below. We performed additional experiments in zebrafish mutants to confirm results obtained from MOs-injected embryos and thoroughly quantified the data as requested. The detailed methods were described in Materials and Methods.

Due to the new data that we have obtained we added several new figures and moved some figures from the supplemental section to the main body of the manuscript, as requested by the reviewers. The revised manuscript includes a total of nine figures, eight supplemental figures, and three movies.

A detailed, point-by-point response to the reviewers' comments and a description of changes made to the manuscript are provided below. Due to the extensive nature of the changes, which were needed to make the language sufficiently precise to get our points across, we did not highlight all changes in the text of the manuscript. For your reference, we have included a version in which the changes are marked using the "compare document" tool in Microsoft Word.

Responses to Reviewer 1:

Major points

1. **Comment**: All reviewers, including Reviewer 1, were concerned about the clarity of the data presented in Figure 1. Particularly, they suggested we include high resolution images and perform "*quantitation across multiple animals to be convincing*".

© 2024. Published by The Company of Biologists under the terms of the Creative Commons Attribution License (https://creativecommons.org/licenses/by/4.0/).

Response: Following the reviewers' suggestions, we obtained high resolution images by repeating the experiments using transgenic lines with relatively high expression levels of the transgenes. We also assessed endoderm organization by analyzing the morphology of endodermal cells on XZ images of confocal Z-stacks.

Cell orientation was determined by measuring the angle of their long axes relative to the mediolateral (ML) axis of the embryos. Consistent with our previous data, our new results demonstrate that endodermal cells initially exhibit mediolateral elongation and gradually reorient towards the ventral side. We present images with improved resolution and descriptions of quantification in the new Figure 2. (Page 7, 178-198)

2. **Comment**: "*further validation with genetic mutants would increase rigor of study (in particular E-cadherin reduction – this should be performed on mutant clutches and scored before genotyping to reduce bias).*"

   Response: We implemented the reviewer's suggestions and conducted immunostaining to assess E-cadherin expression in offspring obtained from in- crosses of *gna13a*$^{-/-}$/*gna13b*$^{+/-}$ mutant fish. We performed confocal imaging and genotyped all embryos after imaging. E-cadherin expression was quantified, with results grouped according to genotype. The findings from the mutants aligned with those observed in embryos injected with MOs. These results are now presented as Figure S8. (Page14, Lines 391-392)

3. **Comment**: "*did not find the p-MLC changes in Fig. 6 and S5 to be particularly convincing, and these lacked any quantitative assessment, sample size, or statistics.*"

   Response: We quantified pMLC localization in both Figs. 6 and S5. These new data are included and are now presented as Figure 5 (Page 13, Line 352-357) and Figure 8 (Page 16, Line 453-455)

4. The reviewer had a few comments on the reduced E-cadherin *in gna13a/b embryos.*
   a) **Comment:** "*It was also unclear why supplying E-cadherin RNA rescued gna13a/b embryos if E-cadherin is regulated at the protein and not transcriptional level. If E-cad levels are regulated at the protein and not transcriptional level, why does providing more mRNA rescue the defect? While rescue of gna13 knockdown by ecad RNA in Fig 5G-J is a nice result, this is again confusing if E- cad levels are controlled at the protein and not mRNA level.*"

      Response: At early stages (e.g., 12s), the E-cadherin protein is uniformly distributed on the plasma membrane of endodermal cells in control embryos, whereas its levels are significantly reduced in *gna13a/gna13b*-deficient embryos (Fig. 6). The injection of *cdh1* RNA leads to ectopic expression of E-cadherin protein, which likely contributes to the rescue of defects that we observed in the *gna13a/gna13b*-deficient embryos. Another potential factor could be that synthetic RNAs lack the 5'UTR and 3'UTR elements of endogenous *cdh1* transcript, which would potentially allow injected RNAs to bypass post-transcriptional regulation and increase protein levels of E-cadherin. We have added this point in the discussion session. (Page 20, Line 570-575)

   b) **Comment:** "*Fig 4J - strong reduction of E-cad protein in whole embryo at 9s is a bit surprising as presumably E-cad is also expressed in many non-endodermal epithelial cells at this stage. What is the explanation for this?*"

      Response: E-cadherin expression is not confined to endodermal cells but extends to mesodermal cells as well, which has been reported in previous studies (Lin et al., 2009). Thus, the observed decrease in E-cadherin expression in whole embryo samples (Fig. 6J) is not necessarily surprising given the uniform expression of *gna13a* and *gna13b* across all germ layers (Lin et al., 2005).

5. **Comment**: "*Fig S5E,J - images of pMLC in controls vs. MOs are not convincing. How consistent are these defects? Needs quantification across multiple embryos.*"

© 2024. Published by The Company of Biologists under the terms of the Creative Commons Attribution License (https://creativecommons.org/licenses/by/4.0/).

Response: We performed quantification of the data, and the results are incorporated into Figure 5. (Page 13, Line 352-357)

Minor points

1. **Comment**: "*While endoderm phenotypes have previously been reported for morpholino knockdown of gna13a/b, a strength of this paper is validation of phenotypes using genetic gna13a/b mutants. This should be moved to the main figure rather than relegated to Figure S4.*"

   Response: The reviewer likely referred to Figure S2, which shows that the mutants displayed the same phenotypes observed in morpholino-injected embryos. We have reassigned Figure S2 as Figure 1. (Page 5-6, Line 120-149)

2. **Comment**: "*The green label in Figure 3 should be stated in legend.*"

   Response: The information for the transgenic line has been included in both the figure and the figure legend.

3. **Comment**: "*…the second (not first) PA is situated between the first and second EP.*"

   Response: Our original text stated: "The first PA is situated between the first and second EP." The reviewer is correct. The mistake has been corrected, and those sentences have been rewritten as "The first PA is located adjacent to the first EP". (Page 12, Line 320).

4. **Comment**: "*Transgenic analysis of myosin-GFP is a strength - Fig S5 should be made a main figure.*"

   Response: We took the reviewer's suggestion and presented these data in Figure 5. (Page 12, Line 328-341)

5. **Comment**: "*N-cadherin data mentioned on p. 17 should be shown.*"

   Response: These data have been presented in Figure S7. (Page 13, Line 369-371)

6. **Comment**: "*Lines 382-383. Should say "Fig. 4K"*.

   Response: Fig. 4K has been reassigned as Figure 6K. The results for Figure 6K are described. (Page 14, Line 389-391).

7. **Comment**: "*In the clonal E-cad MO experiments of Fig. 6, the reduction of ZO1 is convincing but not the reduction of p-MLC. Again, how consistent are these defects? Needs quantification across multiple embryos.*"

   Response: Quantification analyses have been performed, and the results are presented in Fig. 8G, H. (Page 16, Line 449-458)

8. **Comment**: "*Legend to Fig. 7F,G - should say white arrows (not arrowheads).*"

   Response: The error in Fig. 9 has been corrected. (Page 26, Line 758)

9. **Comment**: "*In Methods, it is stated that mem-GFP RNA was used as lineage tracer for endoderm transplants but I don't see this experiment in any figure.*"

   Response: The information on *mem-GFP and H2B-GFP* RNA has been added. (Page 16, Line 438-440; Page 25, Line 733).

10. **Comment**: "*Quantification (at minimum number of embryos examined and how often the shown phenotype was observed) needs to be provided for each experiment. For example Fig. S4 but many others.*"

© 2024. Published by The Company of Biologists under the terms of the Creative Commons Attribution License (https://creativecommons.org/licenses/by/4.0/).

Response: Quantification analyses have been performed for all data presented, including that in the original Figure S4. The new results have been incorporated and presented as Figures 2, 3, 5, 8, S4-8.

Responses to Reviewer 2:

Major points

Comment: "*The main weakness is the lack of quantification provided for the morphogenetic events presented in Figs. 1 and 2. As is, it is unclear how consistent these patterns are between embryos of the same genotype/stage. e.g. How commonly do the "holes" in the pharyngeal sheet appear? Are the changes in nuclear orientation reproducible?*"

Response:

a) The concerns regarding Fig. 1 were also raised by reviewer 1 and were addressed above. Briefly, we obtained high resolution images and assessed endoderm organization by analyzing the morphology of endodermal cells on XZ images of confocal Z stacks. Cell orientation was measured and quantified. Data are now presented in the new Figure 2. (Page 7, Line 178-195)

b) For Figure 2, we have conducted detailed quantitative analyses of the number, area, and duration of the gaps in the pharyngeal sheet. The results have been described and presented in Fig. 3C-F. (Page 8-9, Line 225-242)

Minor points

1. **Comment**: "*Line 43: Change 'the innermost layers of the germ layer" to "the innermost of the germ layers'*"

   c) **Response**: The typo is corrected. (Page 3, Line 49)

2. **Comment**: "*Lines 58-62, these sentences use repeated words.*"

   Response: "These sentences have been rephrased". (Page 3, Line 61-66)

3. **Comment**: "*Line 70: The authors write that 'pharyngeal endoderm cells do not exhibit polarity', in the context of non-canonical Wnt signaling. If they are referring only to planar cell polarity, they should make that explicit; otherwise it is confusing, as this paper describes a role for Ga13 in establishing apicobasal polarity.*"

   Response: The sentence has been rewritten to clearly state that polarity refers to planar cell polarity. (Page 3, Line 71)

4. **Comment**: *Lines 131-132: There is a missing adjective after "exhibited" in the sentence: "Additionally, gna13a-/-/gna13b-/- embryos frequently exhibited heads and eyes". Reduced? Abnormal?*

   Response: The *gna13a*$^{-/-}$/*gna13b*$^{-/-}$ embryos frequently exhibited smaller eyes. We have added this detail to clarify. (Page 5, Line 130)

5. **Comment**: "*Line 314: 'The first PA is situated between the first and second EP' is incorrect - the first endodermal pouch separates the first and second pharyngeal arches. The observed defects in Meckel's cartilage (derived from the first pharyngeal arch) may be attributable not to pouch defects but rather to abnormalities in the more anterior endodermal cells that contact the oral ectoderm.*

   Response: The sentence has been rewritten as "The first PA is located adjacent to the first EP". (Page 12, Line 320)

© 2024. Published by The Company of Biologists under the terms of the Creative Commons Attribution License (https://creativecommons.org/licenses/by/4.0/).

6. **Comment**: *"Fig. 3: Provide labels for the stains/reporters used in this figure."*

   Response: The information on the transgenic lines has been added in the Figure 4.

7. **Comment**: *"Lines 411-412: Please use the gene name for E-cadherin (cdh1) when referring to the RNA injections. And Line 863: Replace 'chd1' with 'cdh1'."*

   Response: The typo referring to *cdh1* has been corrected. (Page 14, Line 389; Page 15, Line 417; Page 29, Line 841)

8. **Comment**: *"Lines 432-434: Please provide more detail in the text on this experiment involving H2B-GFP RNA injections – was this the same protocol as shown in Fig. 5a. (i.e. endoderm transplants), but with this nuclear-targeted GFP used as a marker instead of Rhodamine-dextran?"*

   Response:. Details of the transplantation in this figure have been added to the figure legend and Materials and Methods sections. (Page 16, Line 438-440; Page 25, Line 733)

Responses to Reviewer 3:

Major points

1. **Comment**: "*it is important to confirm that the key phenotypic observations, particularly those made in Figures 3 and 4 (regarding ZO-1 localization and E- cadherin levels and localization) are also apparent in the gna13a;gna13b double homozygotes."*

   Response: Similar comments were raised by reviewer #1. We have conducted additional experiments on *gna13a/gna13b* mutant embryos to complement the data obtained from experiments performed with morpholinos, which validates our initial findings. We have quantified the data and have presented these as Fig. S4 (Page 11, Line 290-291) and Fig. S8 (Page 14, Line 391-392) in the revised manuscript.

2. **Comment**: *All reviewers including this one had similar concerns on the resolution of images shown Figure 1 and quantification.*

   Response: We have acquired high-resolution images and evaluated the organization of the endoderm by examing the morphology of endodermal cells on XZ images obtained from confocal Z stacks. Quantification of cell orientation was performed, and the findings are depicted as Figure 2 of the revised manuscript.

   Detailed descriptions of the data can be found in the text. (Page 7, Line 178-194)

3. **Comment**: "*In Figure 2, while the effect of the MOs on cell-cell contacts is easy to discern, the described differences in cell shapes and orientations are not as clear in the figure, and quantification and/or clarification would help. For example, for the nuclei that are tilting ventrally near the end of the wild-type time-lapse, is the whole cell (and the whole tissue) tilting or just the nucleus?*"

   Response: Similar comments were made by Reviewer #2. We have conducted detailed quantitative analyses of the number, area, and duration of the gaps of the pharyngeal sheet. The results have been described and presented in Fig. 3C-F. (Page 8-9, Line 225-242)

   In terms of the cell shape changes presented in Figure 2, because of the limited number of cells that could be tracked, we conducted these analyses only in fixed embryos at different developmental stages. We obtained high resolution images and assessed endoderm organization by analyzing the morphology of endodermal cells in XZ images from confocal Z stacks. Cell orientation was measured and quantified. The data are now presented in the new Figure 2. (Page 7, Line 178-194). From the images in Figure 2, we observed changes in

© 2024. Published by The Company of Biologists under the terms of the Creative Commons Attribution License (https://creativecommons.org/licenses/by/4.0/).

orientation of the entire cell, including the nucleus.

4. **Comment**: "*In Figure S5, the contrast between organized and disorganized myosin- GFP localization is clear, but the relative concentration on the apical surface is less evident in the images. Quantification of this localization, similar to that performed for ZO-1 in Figure 3, would be valuable.*"

Response: New experiments were conducted, and quantification of myosin-GFP localization in 10s-embryos was performed and is presented in Fig. 5A-C of the revised manuscript (Page 12, Line 331-341). Regarding the relative concentration of myosin- GFP, it is unreliable to quantify the intensity of myosin-GFP using the transgenic lines because the copy number of the transgene in the genome may vary between embryos.

5. **Comment**: "*The results shown in Figure 5G-K don't seem to make sense — if E- cadherin transcript levels are normal in the gna13-deficient embryos, then why does injection of cdh1 RNA rescue endoderm width?*"

Response: Reviewer #1 had similar comments. At early stages (e.g., 12s), the E- cadherin protein is uniformly distributed on the plasma membrane of endodermal cells in control embryos, whereas its levels are significantly reduced in *gna13a/gna13b*-deficient embryos (Fig. 6). The injection of *cdh1* RNA leads to ectopic expression of E-cadherin protein, which likely contributes to the rescue of defects that we observed in the *gna13a/gna13b*-deficient embryos. Another potential factor could be that synthetic RNAs lack the 5'UTR and 3'UTR elements of endogenous *cdh1* RNA, which would potentially allow injected RNAs to bypass post-transcriptional regulation and increase protein levels of E-cadherin. We have added this point in the discussion session. (Page 20, Line 570-575)

Minor points

1. **Comment**: "*While the manuscript is generally clearly written, it would be beneficial for the authors to proofread carefully for typos, examples of which include: 'Wnt111r' should be 'Wnt11r', 'alternations' should be 'alterations', 'titled' should be 'tilted', etc. Also, some language can be simplified: for example, it is not necessary to say 'genetic mutant' or 'genetic mutations', since 'mutant' and 'mutations' are sufficient to convey the appropriate meaning.*

Response: We carefully proofread the manuscript and corrected any typos.

2. **Comment**: "*Perhaps this view is too old-fashioned, but it would be preferable to discuss ZO-1 'localization' rather than 'expression' when describing the results in Figure 3, reserving 'expression' for the description of transcriptional regulation and 'localization', 'levels', 'presence', etc., to describe where proteins are found. Similarly, it would be helpful to change the semantics to discuss 'localization', 'levels', 'presence', etc., of E-cadherin protein and 'expression' of E-cadherin transcripts.*"

Response: We agree that using the terms "localization of ZO-1 and E-cadherin" better describes the phenotypes we observed. All text has been modified accordingly.

Lin, F., Chen, S., Sepich, D. S., Panizzi, J. R., Clendenon, S. G., Marrs, J. A., Hamm, H. E. and Solnica-Krezel, L. (2009). G¤12/13 regulate epiboly by inhibiting E-cadherin activity and modulating the actin cytoskeleton. *J Cell Biol* **184**, 909-921.
Lin, F., Sepich, D. S., Chen, S., Topczewski, J., Yin, C., Solnica-Krezel, L. and Hamm, H. (2005). Essential roles of G¤12/13 signaling in distinct cell behaviors driving zebrafish convergence and extension gastrulation movements. *J Cell Biol* **169**, 777-787.

Second decision letter

© 2024. Published by The Company of Biologists under the terms of the Creative Commons Attribution License (https://creativecommons.org/licenses/by/4.0/).

MS ID#: DEVELOP/2023/202597

MS TITLE: Gα13 controls pharyngeal endoderm convergence by regulating E-cadherin expression and RhoA activation

AUTHORS: bo hu, Joshua Pinzour, Asmi Patel, Faith Rooney, Amie Zerwic, Yuanyuan Gao, Nhan T Nguyen, Huaping Xie, Ding Ye, and Fang Lin

You will be pleased to read that the reviewers are happy with your revisions and there are just very minor comments to consider before publication. The referees' comments are appended below, or you can access them online: please go to BenchPress and click on the 'Manuscripts with Decisions' queue in the Author Area.

Reviewer 1

Advance summary and potential significance to field

The authors have satisfactorily addressed all my concerns.

Comments for the author

No further revisions are required in my view.

Reviewer 2

Advance summary and potential significance to field

In this study, Hu et al. investigate mechanisms controlling early morphogenesis of the pharyngeal endoderm in zebrafish embryos, preceding the formation of endodermal pouches. This group previously reported roles for Ga13a and Ga13b in pharyngeal endoderm convergence, myocardial cell migration, and caudal fin formation, using morpholino knockdowns (Ye and Lin, 2013). Here they probe deeper using genetic loss of function models, finding that deficiency of Ga13 proteins interferes with medial convergence, rosette formation, and cell-cell adherence of the pharyngeal endoderm sheet, though not formation of endodermal pouches. Using a clever set of transplant and rescue experiments, they tie convergence phenotypes to reduced levels of E-cadherin protein and disruption of cytoskeletal changes associated with apical constriction. Some but not all of these processes appear to be mediated by RhoA downstream of Ga13a.

Beyond the Ga13a studies, the authors also provide a wealth of data on the basic biology of the endodermal convergence process, which will be of value for the field. Overall, this well-controlled, carefully-quantified study investigates an essential early step in endodermal development that organizes the tissue for subsequent organ development.

Comments for the author

The concerns I raised in my first review have been thoroughly addressed. The added quantification of the phenotypes shown in the new Figs. 2 and 3 lends credence to the authors' conclusions and has strengthened the manuscript.

Very minor:
The craniofacial phenotypes shown in Fig. S6 (and lines 319-320) are very striking, and the morphometric measurements are plausible. However, it is confusing to refer to the second measurement as the “length of the lower jaw”. Meckel’s cartilage is the functional lower jaw of fish larvae, and it is measured separately. The authors define this second measurement as “the distance from the anterior end of Meckel’s cartilage to the hyosymplectic cartilage” – this also encompasses the length of the palatoquadrate, which is part of the jaw apparatus but not

© 2024. Published by The Company of Biologists under the terms of the Creative Commons Attribution License (https://creativecommons.org/licenses/by/4.0/).

specifically the lower jaw. I recommend changing the name of this measure to something like “length of the jaw skeleton” or “length of the first-arch-derived skeleton”.

Line 419: change chd1 to cdh1

Reviewer 3

Advance summary and potential significance to field

The authors have done a careful and thorough job of addressing the reviewers' comments, and the revised manuscript will be valuable to the readers of Development.

Comments for the author

The authors have done a careful and thorough job of addressing the reviewers' comments, and the revised manuscript will be valuable to the readers of Development.

Second revision

Author response to reviewers' comments

Overall response:

We thank the reviewers for recognizing our efforts to address all the issues raised and for acknowledging the thoroughness of our revisions.

Responses to Reviewer 2:

Minor points

1. Comment: The authors define this second measurement as “the distance from the anterior end of Meckel’s cartilage to the hyosymplectic cartilage” – this also encompasses the length of the palatoquadrate, which is part of the jaw apparatus but not specifically the lower jaw. I recommend changing the name of this measure to something like “length of the jaw skeleton” or “length of the first-arch-derived skeleton”.
Response: We took the reviewer's suggestion and used the term “the jaw skeleton” instead of “the lower jaw”.

2. Comment: Line 419: change chd1 to cdh1.
Response: The typo is corrected.

Third decision letter

MS ID#: DEVELOP/2023/202597

MS TITLE: Gα13 controls pharyngeal endoderm convergence by regulating E-cadherin expression and RhoA activation

AUTHORS: bo hu, Joshua Pinzour, Asmi Patel, Faith Rooney, Amie Zerwic, Yuanyuan Gao, Nhan T Nguyen, Huaping Xie, Ding Ye, and Fang Lin
ARTICLE TYPE: Research Article

I am happy to tell you that your manuscript has been accepted for publication in Development, pending our standard publication integrity checks.

© 2024. Published by The Company of Biologists under the terms of the Creative Commons Attribution License (https://creativecommons.org/licenses/by/4.0/).
